# Gelatin-Based Rapid Blue Light-Irradiation In Situ Gelation Hydrogel Platform for Combination Therapy in Brain Tumors

**DOI:** 10.3390/pharmaceutics17101353

**Published:** 2025-10-20

**Authors:** Chiung-Yin Huang, Hung-Wei Yang, Hung-Chun Wang, Chia-Yu Hsu, Kuo-Chen Wei, Pin-Yuan Chen, Hao-Han Pang

**Affiliations:** 1 Neuroscience Research Center, Chang Gung Memorial Hospital, Linkou, Taoyuan 33305, Taiwan; 2 Department of Biomedical Engineering, National Cheng Kung University, Tainan 70101, Taiwan; 3 Medical Device Innovation Center, National Cheng Kung University, Tainan 70101, Taiwan; 4 Institute of Medical Science and Technology, National Sun Yat-sen University, Kaohsiung 80424, Taiwan; 5 Center for Drug Research and Development, Collage of Human Ecology, Chang Gung University of Science and Technology, Taoyuan 33303, Taiwan; 6 Department of Neurosurgery, Chang Gung Memorial Hospital, Linkou, Taoyuan 33305, Taiwan; 7 School of Medicine, Chang Gung University, Guishan, Taoyuan 33302, Taiwan; 8 Department of Neurosurgery, Chang Gung Memorial Hospital, Keelung 20401, Taiwan

**Keywords:** glioblastoma, GBM, combination therapy, hydrogel, photothermal therapy

## Abstract

**Background/Objectives**: Glioblastoma (GBM) is a fatal tumor in the central nervous system (CNS) with a poor prognosis. Preventing tumors from post-surgical recurrence is a significant clinical challenge, since current methods deliver chemotherapeutic agents in a rapid manner and are not effective against the residual tumor cells. To address these limitations, we develop a blue light-crosslinking hydrogel which can be rapidly gelled in situ and tightly adhere on the tissues for controlled chemotherapy, radiotherapy, and enhanced laser interstitial thermal therapy (LITT) to inhibit residual tumor cells from post-surgical recurrence. **Methods**: We utilize gelatin-MA based hydrogel with crosslinker VA-086 as hydrogel scaffold to encapsulate small-molecule drugs (Epirubicin and Cisplatin) and LITT agent polypyrrole-coated graphine oxide (PPy@GO). The mixture can form into hydrogel in situ by blue light irradiation and performed chemo-LITT and radio therapy simultaneously. Then we determine the prevailing factors that affect efficient encapsulation of therapeutic agents within hydrogels, efficiency of gelation, LITT enhancement, and drug release. Then evaluate efficiency in human cancer cells and an in vivo tumor model. **Results**: Our results demonstrate that 18 wt% Gelatin MA formulation achieved >95% gelation within 2 min, with drug-loaded gels forming within 5 min. The gelation can perform both in vitro and in vivo without affect the drug efficiency. This multi-treatment system can effectively prevent tumor recurrence and significantly prolong the medium survival of glioma-bearing (MBR-614 or U87-MGFL) mice to above 65 days compared with the control group (36 days). **Conclusions**: The results demonstrated promising effect of this system as a multi-therapeutic platform which combined chemo-LITT and RT. This synergistic strategy presents a new approach to the development of a local drug delivery system for the prevention of brain tumor recurrence.

## 1. Introduction

Glioblastoma (GBM) is one of the most fatal tumors in the central nervous system. The average survival of GBM is lower than 24 months and the 5-year survival rate is lower than 10% [1,2,3,4]. Even with the tumor being eliminated by therapy, the median recurrence time of GBM is lower than 1 year. Current therapy methods such as surgery, radiotherapy (RT), and chemotherapy are not efficient enough due to the resistance of tumor cells and natural biological barriers like the blood–brain barrier (BBB). The surgical removal of tumor cells cannot remove all tumor cells that have already invaded normal tissues or metastasized [5]. Most recurrent tumor cells will show higher resistance to RT or chemotherapy [6]. The BBB will also block most of drugs. For example, the rate of the most used drug, temozolomide (TMZ), that can approach tumors from the blood stream is lower than 20% [3,7,8]. Therefore, there is a critical need to develop a combined therapy method that can overcome these challenges to improve the treatment outcome for GBM patients.

Cisplatin is a platinum-based chemotherapeutic agent that exerts its cytotoxic effects primarily by forming intra- and inter-strand DNA crosslinks, particularly at guanine–cytosine sites. This action disrupts DNA replication and transcription, ultimately triggering cell cycle arrest and apoptosis [9]. Furthermore, cisplatin enhances oxidative stress within cancer cells by impairing mitochondrial function [10,11], and its potent radiosensitizing properties [12], stemming from the Auger electron effect of the platinum atom, make it an attractive candidate for combined modality treatment in glioblastoma (GBM). Epirubicin (EPI) is an anthracycline antibiotic and an epimer of doxorubicin (DOX). EPI shares a similar mechanism with DOX, involving DNA intercalation and the inhibition of topoisomerase II, which also obstructs nucleic acid and protein synthesis [13]. Crucially, EPI was developed to offer a superior therapeutic index, exhibiting significantly lower cardiotoxicity than its predecessor while maintaining comparable efficacy [14]. The combination of cisplatin and EPI is already established in clinical practice for various systemic cancers, and given that both agents are DNA-damaging drugs with demonstrated potential in treating gliomas, their co-delivery presents a synergistic strategy [15,16,17]. However, their systemic administration is hampered by severe side effects, including nephrotoxicity and neurotoxicity for cisplatin [18] and dose-cumulative cardiotoxicity and myelosuppression for EPI [19]. These highlight the critical need for a localized and sustained-release delivery system to enhance therapeutic outcomes in GBM while minimizing systemic toxicity.

Photothermal therapy (PTT) is a potential therapy method to treat brain tumors due to its non-invasive and high efficiency in eliminating tumor cells [20,21]. PTT will need a photosensitizer and a delivery method for the photosensitizer and light to the tumor site. PTT brings the following benefits. First, the excitation wavelength of PTT needed is generally near-infrared (NIR), which has a longer wavelength, deeper tissue penetration, and, therefore, is less harmful to normal cells and tissues. Another benefit is that the efficiency of killing cancer cells is high. Wei Lu and coworkers demonstrated that by using gold nano particles with 808nm laser, the local temperature could reach 54 °C within 3 min, causing cell death for tumors and enhancing the overall survival in vivo [22]. Therefore, PTT can be a promising therapy method for GBM. Polypyrrole (PPy) is a polymer that widely used in PTT because of its photothermal activity [23,24]. Graphene oxide (GO) is a two-dimensional carbon nanomaterial that is widely used in biomedical applications [25,26,27,28,29,30]. With a large surface area (2630 m^2^/g) and enriched oxygen-containing reactive groups such as COOH and OH groups, GO can be an ideal delivery platform for conjugating/carrying drugs, DNAs, and polymers [31,32]. GO is also a promising material for PTT in cancer [33,34]. In this study, we utilized Polypyrrole-coated graphene oxide (PPy@GO) as a photosensitizer to enhance PTT efficiency.

Controlled-release delivery technologies for drug delivery are also important in cancer therapy, especially in brain tumors. Normal brain tissues are sensitive to toxic drugs or high-energy therapy like PTT. Therefore, it is necessary to control the drugs and photosensitizers to stay at the target site to enhance the local concentration of drugs and reduce side effects. Among delivery strategies, hydrogel is a promising material for overcoming this challenge. Hydrogels are three-dimensional, water-rich polymer networks that are ideal for controlled and localized drug release. Because of its high biosafety and biocompatibility, hydrogel is used in many therapeutic applications [35,36]. Gelatin is a popular biomaterial as the base polymer of hydrogel. However, Gelatin is in liquid form during application without crosslinking. Therefore, in this study, we utilized 2,2′-Azobis [2-methyl-*N*-(2-hydroxyethyl)propionamide] (VA-086) as a blue-light-triggered photo-polymerization initiator that induces polymorization of gelatin and maleic acid (Gel-MA) [37]. The activation wavelength of VA-086 is within visible light range, which reduces the risk for UV exposure. The Gel-MA hydrogel has high biocompatibility and low toxicity. These advantages make VA-086-initiated Gel-MA hydrogels an ideal material for local controlled-release delivery methods with just a short time of light irradiation.

**Scheme 1:**
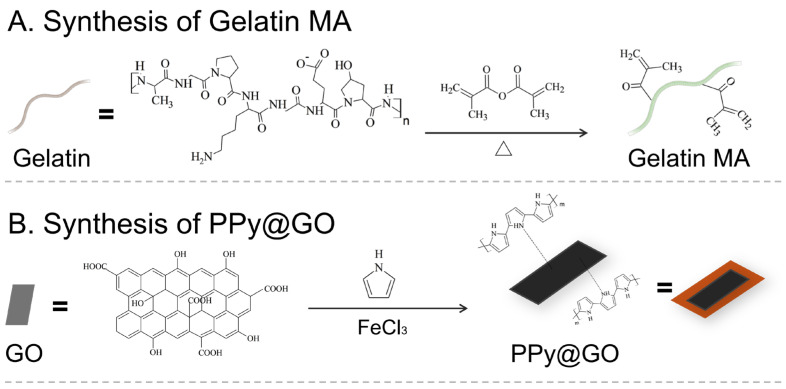

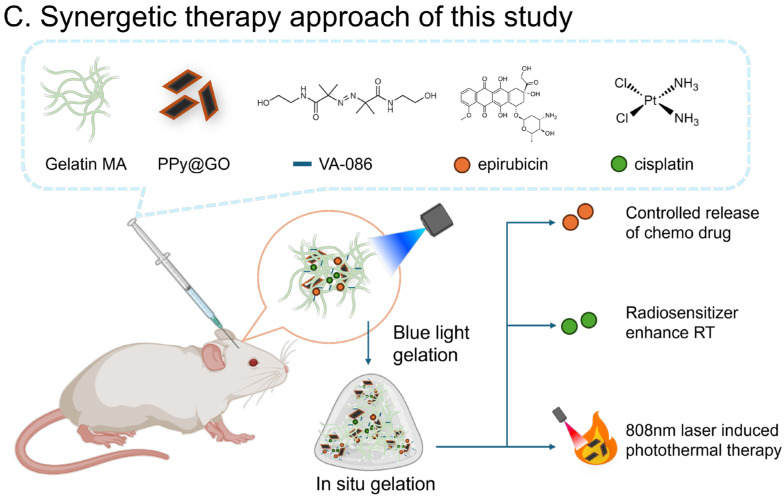
Schematic illustration of the overall design of the study. (**A**) Gelatin was modified to Gelatin-MA, and (**B**) graphene oxide was coated with polypyrrole (PPy@GO). (**C**) These components, together with therapeutic drugs (epirubicin and cisplatin) and a photosensitizer, were mixed to form a hydrogel. Upon blue-light irradiation, the hydrogel was crosslinked in situ. Subsequent exposure to 808 nm near-infrared light enabled intratumoral gelation, photothermal heating, and chemo–photothermal combined cancer therapy.

Brain tumors are aggressive and easily develop resistance against therapy. Therefore, combination therapy is necessary to eradicate brain tumors. Therefore, we aimed to develop a multi-functional hydrogel-based delivery system that can deliver different kinds of chemotherapy drugs (cisplatin and epirubicin) with PPy@GO as a PTT photosensitizer simultaneously, as shown in Scheme 1. The goals of this combination design are as follows. First, to develop a biocompatible material that can deliver chemotherapy agents directly to the brain. Second, to enable controlled and sustained drug release at the tumor site, thereby reducing systemic side effects. Third, to utilize the photothermal effect to directly kill tumor cells and enhance the efficacy of chemotherapy. Fourth, to achieve in situ polymerization in vivo with minimal adverse effects. These goals aim to address the shortcomings of current GBM treatments and offer a more effective therapy method to enhance GBM therapy. The demonstration of this novel combination across PTT, RT, and chemotherapy platforms will be a proof-of-concept model for the combination therapy of GBM in the future.

## 2. Materials and Methods

### 2.1. Hydrogel Preparation

#### 2.1.1. Gelatin Modification and Purification

Gelatin modified with methacrylate groups was used as the material for hydrogel preparation. Gelatin was dissolved in PBS buffer solution (5 mM, pH 7.4) with continuous heating and stirring to obtain a 10% (*w*/*w*) gelatin solution. The solution was heated to 60 °C, and 3.2 mL of methacrylate anhydride was added dropwise under vigorous stirring for 2 h (light-protected). The reaction was terminated by adding 200 mL cold PBS (pH 7.4). The resulting cloudy product was diluted with PBS and transferred into a dialysis bag, sealed with rubber bands. The bag was immersed in deionized water (D.I. H_2_O) at 40 °C, with the water replaced every 4–6 h for three days. After dialysis, the clear product was collected. If precipitate was present, the mixture was centrifuged at 4000 rpm for 30 min, and the supernatant was collected as the pale-yellow transparent Gelatin MA solution. The solution was concentrated with a rotary evaporator until an orange-yellow transparent viscous liquid was obtained. It was then freeze-dried using a lyophilizer to obtain a dried ivory-white Gelatin MA powder.

#### 2.1.2. Synthesis of Photothermal Material

##### Synthesis of Bovine Serum Albumin@Graphene (BSA@GO)

Bovine serum albumin (BSA, 200 mg) was dissolved in deionized water (D.I. H_2_O) and the pH was adjusted to 3.6. Graphite (4 g) was added, and the total volume was adjusted to 200 mL. The suspension was subjected to probe sonication for 2 h with intermittent ice-bath cooling to maintain the temperature below 40 °C. The dispersion was stored at 4 °C overnight to allow sedimentation, after which the supernatant was decanted. The product was collected by centrifugation at 12,000 rpm, washed several times with D.I. H_2_O, and re-dispersed in a fixed volume of D.I. H_2_O. The sample was subsequently lyophilized to obtain BSA/GO powder, which was stored at −20 °C until use. For experiments, the lyophilized product was re-dissolved in sterilized D.I. H_2_O at a working concentration of 8 mg/mL.

##### Synthesis of Polypyrrole@GO

Graphene oxide (GO) was synthesized using a modified Hummers’ method. Briefly, graphite (1 g) and sodium nitrate (1 g) were added to concentrated sulfuric acid (45 mL) and stirred in an ice bath for 4 h. Potassium permanganate was slowly introduced while maintaining the temperature below 15 °C. The mixture was diluted with deionized water (100 mL) and stirred for 2 h, followed by heating at 35 °C for 2 h and subsequently at 98 °C for 10 min. Additional deionized water (30 mL and then 50 mL) was added in sequence, with stirring for 1.5 h and 1 h, respectively. The product was collected by centrifugation (10,000 rpm, 15 min) and repeatedly washed with 0.1 M HCl and 0.1 M NaOH solutions, followed by centrifugation and resuspension, until the supernatant reached near-neutral pH, yielding purified GO.

For PPy@GO synthesis, GO (5 mL, 1 mg/mL) was mixed with polyvinylpyrrolidone (PVP, 100 mg) in deionized water (50 mL) and stirred at room temperature for 2 h. Pyrrole monomer (8 μL) was then added, and stirring continued for another 2 h. Ferric chloride (FeCl_3_, 50 mg) was subsequently introduced as an oxidant, and the mixture was stirred for 24 h, producing a black dispersion. The product was purified by centrifugation (12,000 rpm) and repeated washing with deionized water, with redispersion by ultrasonication. The final suspension was collected and quantified to obtain PPy@GO.

#### 2.1.3. Preparation of Drug and Material Solutions:

Cisplatin powder was dissolved in 0.05 M HCl with ultrasonication to yield a 5 mg/mL stock solution. Epirubicin (EPI) was dissolved in deionized water (D.I. H_2_O) using ultrasonication and vortexing to obtain an 80 mg/mL solution. PPy@GO was prepared as a 500 μg/mL dispersion. VA-086 was suspended in D.I. H_2_O at 200 mg/mL in light-protected amber tubes.

Lyophilized Gelatin MA was reconstituted by dissolving the powder in D.I. H_2_O at 70 °C with intermittent shaking to obtain a 25% (*w*/*w*) solution. Aliquots of this solution were then mixed with cisplatin (5 mg/mL), EPI (80 mg/mL), PPy@GO (500 μg/mL), VA-086 (200 mg/mL), and D.I. H_2_O according to the formulations listed in Table 1. The mixtures were incubated in a 60 °C dry bath for 5 min under light-protected conditions and subsequently vortexed to ensure homogeneity.

#### 2.1.4. Light-Initiated Gelation

Gelatin MA and the designated materials were combined in a microcentrifuge tube and homogenized by vortex mixing. The mixture was incubated in a 60 °C dry bath, followed by the addition of VA-086 suspension (250 mg/mL) in the prescribed amount (Table 1) and further vortexing. After 1–2 min, when the mixture became transparent, the samples were irradiated with 395 nm blue light (3 W, 2–4 cm distance) for 5 min, resulting in hydrogel formation.

### 2.2. Characterization and Analysis of Hydrogel

#### 2.2.1. Fourier Transform Infrared Spectroscopy (FTIR) and Nuclear Magnetic Resonance (NMR) Spectroscopy

Dry Gelatin and Gelatin MA were ground into a fine powder, mixed with KBr (solid), and then pressed into pellets. FTIR analysis was carried out using a Fourier Transform Infrared Spectrometer over the wavenumber range of 400–4000 nm. The data were processed according to the relationship between absorbance (A) and transmittance (T), where A = −log (T). For hydrogen nuclear magnetic resonance (^1^H NMR) spectroscopy, the dried Gelatin and Gelatin MA were dissolved in D_2_O and loaded into glass NMR sample tubes. Measurements were conducted at a set temperature of 50 °C using ^1^H NMR spectroscopy.

#### 2.2.2. Scanning Electron Microscopy (SEM)

Gelatin MA and VA-086 (ratios shown in Table 1) were mixed and irradiated with blue light for 5 min to induce gelation. Aliquots (5 μL) of the mixture were placed onto 0.4 × 0.8 cm aluminum plates, freeze-dried, and examined by scanning electron microscopy (SEM). For fracture surface analysis, the freeze-dried gels were carefully detached with a blade, fractured, and mounted on aluminum plates with quick-drying adhesive, with the fracture surface oriented upward for SEM observation.

#### 2.2.3. Gelation Time Study

Equal volumes of Gelatin MA and VA-086 (Table 1 ratio) were transferred into pre-weighed 0.5 mL microcentrifuge tubes. After recording the weight of the mixture, samples were irradiated with blue light for 0.3, 0.5, 1, 1.5, 2, 3, or 5 min (seven groups). At each time point, un-gelled liquid was removed with a micropipette, the gels were washed with deionized water, and residual liquid was extracted. The tubes containing the remaining gels were reweighed, and the percentage of gelation was calculated and plotted as a function of irradiation time.

Weight of hydrogel and photosensitizer = Weight of tube with hydrogel − Weight of empty centrifuge tube

Weight of gel = Weight of tube with gel − Weight of empty centrifuge tube

$$Gel\;fraction\;(\%)=\frac{Weight\;of\;gel}{Weight\;of\;hydrogel\;and\;photosensitizer}\times 100\%$$

#### 2.2.4. Thermogravimetric Analysis (TGA)

Prepare 0.5 g each of gelatin powder, gelatin methacryloyl (GelMA), and lyophilized hydrogel (completely dried using a vacuum dryer) by grinding them into powder form. Subject each sample to thermogravimetric analysis (TGA) with a heating rate of 20 °C/min, recording the remaining weight of the sample every 0.5 s. Continue heating until reaching 800 °C, and monitor the weight loss. Plot the percentage of residual mass (defined as the remaining weight of the sample divided by its initial weight, multiplied by 100%) against temperature. Furthermore, plot the derivative of the residual mass percentage with respect to temperature. Determine the degradation temperature based on the peak values of the derivative plot.

### 2.3. Photothermal Material Characterization and Analysis

#### 2.3.1. Absorption Spectrum of PPy@GO

PPy@GO and GO dispersions were prepared in deionized water (D.I. H_2_O) and transferred into spectrophotometer cuvettes. Absorption spectra were recorded using a UV–Vis spectrophotometer over the range of 400–900 nm with a scanning speed of 200 nm/min and a data interval of 1 nm. The spectra were analyzed and compared with the reported literature to confirm the successful modification of GO with PPy.

#### 2.3.2. Transmission Electron Microscopy (TEM) Imaging

Aliquots (5 μL) of PPy@GO and GO dispersions (1 mg/mL) were deposited onto 200-mesh copper grids and dried overnight. Samples were examined by transmission electron microscopy (TEM) at an accelerating voltage of 120 kV.

#### 2.3.3. Evaluation of Photothermal Property

Hydrogels were prepared by mixing Gelatin MA, PPy@GO, and VA-086 according to the formulations in Table 1, yielding Gel (without PPy@GO) and P-gel (with PPy@GO). Aliquots (100 μL) were transferred into 1.5 mL microcentrifuge tubes, heated to 60 °C, homogenized, and irradiated with blue light for 5 min to induce gelation. The tubes were then inverted so that the gels were positioned at the top and irradiated with an 808 nm laser (75% output). An FLIR infrared camera was calibrated, and after equilibration to room temperature, sample temperatures were recorded for 5 min during irradiation. Temperature–time curves were generated from the recordings.

For repeated heating experiments, 200 μL of Gel or P-gel was deposited on glass slides and irradiated with an 808 nm laser (75% output) for 90 s, followed by cooling for 210 s. This cycle was repeated three times. Sample temperatures were monitored in real time using an FLIR infrared camera.

#### 2.3.4. In Vitro Cytotoxicity of PPy@GO

Hydrogels were prepared by mixing Gelatin MA, PPy@GO, and the photosensitizer in the ratios listed in Table 1. Aliquots (25 μL) were evenly spread at the bottom of wells in a 96-well cell culture plate and irradiated with blue light for 5 min to induce complete gelation. The plates were placed in a biosafety cabinet with airflow activated and left overnight to allow complete drying of the hydrogels. The following three groups were established: P-gel, Gel, and a control group, each comprising 16 wells. Each group was subdivided into irradiated and non-irradiated subgroups (*n* = 8 wells each).

U87-MGFL cells were seeded at 9000 cells per well in DMEM medium (final volume 210 μL per well) and incubated for 24 h to allow attachment and stabilization. Experimental groups were irradiated with an 808 nm near-infrared (NIR) laser at 75% output for 10 min, after which the plates were returned to the incubator. After 24 h, the medium was removed, wells were washed with PBS (pH 7.4), and cells were treated with phenol-red-free DMEM–XTT salt (1 mg/mL)–PMS (3 mg/mL in PBS) in a ratio of 100:50:1 (120 μL per well) under light-protected conditions. After 2 h of incubation, 100 μL of supernatant was transferred to a fresh 96-well ELISA plate, and absorbance was measured at 450 nm. For each group, the highest and lowest readings were excluded, and the mean of the remaining six wells was used to calculate relative cell viability (%), defined as (mean absorbance of experimental group ÷ mean absorbance of control group) × 100.

Statistical significance was evaluated using the built-in *t*-Test function in Microsoft Excel (two-tailed, type 3: unequal variance). A *p*-value of < 0.05 was considered statistically significant, and *p* < 0.001 highly significant.

### 2.4. Drug Release and Cytotoxicity of Drug-Loaded Hydrogels

#### 2.4.1. EPI Release Profile

To assess drug release, epirubicin (EPI) was selected as the model drug due to its measurable absorption at 395 nm. Standard EPI solutions (0.005–0.5 mg/mL) were analyzed by UV–Vis spectrophotometry at 395 nm to generate a calibration curve (R^2^ = 0.9994). Gelatin MA, EPI, and VA-086 were mixed, heated until homogeneous, and 100 μL aliquots were dispensed into the concave lids of microcentrifuge tubes. Samples were irradiated with blue light for 5 min to achieve gelation, then rinsed with deionized water (D.I. H_2_O) to remove residual surface EPI. Each hydrogel was placed in a 1.5 mL HPLC vial containing 1 mL of PBS (pH 6 or pH 7.4), or PBS at pH 6 supplemented with 500 ng/mL collagenase. Samples were incubated at 37 °C with shaking (140 rpm) and collected at 1, 7, 12, 24, 48, and 72 h. At each time point, the release medium was replaced with fresh PBS, and the collected medium was measured at 395 nm. EPI concentrations were calculated from the calibration curve, and release percentages were determined relative to an aqueous EPI solution of an equivalent concentration to that in the E-gel group.

#### 2.4.2. Cytotoxicity Assay

Gelatin MA, VA-086, and the respective materials were combined at 60 °C in the ratios shown in Table 1 to prepare the following six hydrogel formulations: Gel, C-Gel, E-Gel, CE-Gel, P-Gel, and CEP-Gel. Aliquots (5 μL) were adhered to the side walls of wells in 96-well plates and irradiated with blue light for 5 min to induce gelation. Plates were placed in a biosafety cabinet with airflow on and left overnight for complete drying. Four rows per plate were reserved as untreated controls, and each treatment group included eight replicates. U87-MGFL or MBR-614 cells were seeded at 9000 cells/well and incubated for 8 h before adding DMEM to a final volume of 210 μL/well. For free-drug control groups, equivalent concentrations of cisplatin, EPI, or cisplatin + EPI solution were added directly in solution form.

At 12, 24, 48, and 72 h, medium was removed, and wells were rinsed with 100 μL PBS (pH 7.4). Fresh phenol-red-free DMEM, XTT salt (1 mg/mL), and PMS (3 mg/mL in PBS) were mixed (100:50:1), and 120 μL of this solution was added per well under light-protected conditions. After 2 h of incubation, 100 μL of supernatant was transferred to a new plate, and absorbance was measured at 450 nm. For each group, the highest and lowest values were excluded, and the mean of the remaining six replicates was normalized to the untreated control to calculate percentage cell viability.

#### 2.4.3. Fluorescence Imaging

Gelatin MA, EPI, and the photosensitizer (ratios as described in Table 1) were mixed at 60 °C until homogeneous. A 40 μL aliquot was transferred to a 0.5 mL microcentrifuge tube and irradiated with blue light for 5 min to form E-gel. The hydrogel was rinsed with deionized water (D.I. H_2_O) to remove residual surface EPI and exposed to UV light in a biosafety cabinet for several minutes to eliminate potential biological contaminants.

U87-MGFL or MBR-614 cells were seeded in 6-well plates at 2.4 × 10^5^ cells/well in 2 mL DMEM medium and incubated for 24 h to allow attachment. Sterile tips were used to transfer intact E-gel samples into the wells; any fractured gels were discarded. The gels remained in contact with cells for 1, 3, 5, 7, or 12 h, after which they were removed and wells were washed with 2 mL PBS (pH 7.4).

Cells were stained under light-protected conditions with Hoechst 33342 (1:250 dilution in phenol red-free DMEM, 0.5 mL per well) for 10 min. Wells were washed three times with PBS, and 1 mL phenol red-free medium was added before imaging. Fluorescence microscopy was performed under pre-warmed conditions. Cell nuclei were visualized using UV excitation (Hoechst signal), followed by EPI fluorescence imaging at the appropriate green channel absorption wavelength. Imaging parameters were standardized by first focusing, then acquiring images with an exposure time of 220 ms and enhancement factor of 5. Images were overlaid to compare nuclear and EPI fluorescence distribution. Control samples consisted of wells treated with aqueous EPI solution (40 μL D.I. H_2_O, matched to the concentration and volume in the E-gel group). Controls underwent the same incubation, staining, and imaging protocol, with observations taken at the 1 h time point.

### 2.5. Animal Experiments

The animal experiments followed the guidelines from Chang Gung University Institutional Animal Care and Use Committee. The IACUC approval NO. is CGU110-034 (2021/07–2024/06). The animals were purchased from BioLASCO, Taipei City, Taiwan (AAALAC International awarded company). Detailed information for animal experiments and procedures is described in Supplementary Materials Methods S1–S9. Brief descriptions of the main experiments are provided as follows.

#### 2.5.1. In Vivo Gelation of Gelatin MA

C57BL/6 mice were anesthetized with isoflurane using an anesthesia machine. A mixture of Gelatin MA, photosensitizer, and FITC fluorescent dye (FITC at 5% of the final volume) was heated and mixed in a 1 mL microcentrifuge tube. A 40 μL aliquot was drawn into a syringe and injected subcutaneously. Immediately after injection, the site was irradiated with a 395 nm blue-light flashlight at close range for 10 min to induce gelation. Control mice received the same injection without blue-light irradiation. Following treatment, the injection site tissue was excised, and FITC fluorescence was visualized by excitation with blue light for photographic documentation.

#### 2.5.2. In Vivo Photothermal Effect Study

C57BL/6 mice were anesthetized with isoflurane. Gel and P-gel formulations containing the photosensitizer (ratios as described in Table 1) were prepared in 1.5 mL microcentrifuge tubes by heating and thorough mixing. A 40 μL aliquot was loaded into an insulin syringe and injected subcutaneously. The injection site was immediately irradiated with a 395 nm blue-light flashlight at close proximity for 10 min to ensure gelation. Residual gel or materials on the skin surface were removed using 70% ethanol. An 808 nm near-infrared (NIR) laser was then directed at the subcutaneous gel sites, with the output set to 75%. An FLIR infrared camera was calibrated and used to record video and measure local temperature. After confirming the animal and environment had returned to room temperature, NIR irradiation was initiated. Each group was exposed for 5 min, and temperature changes were recorded as a function of irradiation time.

#### 2.5.3. Tumor Suppression Ability of Drug-Loaded Hydrogel In Vivo

C57BL/6 mice were anesthetized with isoflurane using an anesthesia machine. U87-MGFL cells were harvested by trypsinization, centrifuged in DMEM medium, resuspended in FBS, and injected subcutaneously into the ventral side of each mouse (2 × 10^5^ cells per mouse).

On the fifth day after inoculation, when tumors reached approximately 20 mm^3^, treatments were initiated. The initial tumor volume and body weight of each mouse were recorded. The study was terminated upon either the death of a mouse or when the tumor volume reached 2000 mm^3^, which was considered the endpoint.

$$Tumor\;Volume\;Calculation=\frac{Tumor\;length\times Tumor\;width\times Tumor\;height}{2}\;(unit:{mm}^{3})$$

$$Relative\;Tumor\;Volume\;Calculation=\frac{Tumor\;volume\;on\;measurement\;day-Initial\;tumor\;volume}{Initial\;tumor\;volume}\times 100\%$$

$$Relative\;Body\;Weight\;Calculation=\frac{Body\;weight\;on\;measurement\;day-Initial\;body\;weight}{Initial\;body\;weight}\times 100\%$$

For hydrogel-based therapy, P-gel and CEP-gel were prepared according to the ratios in Table 1, while Freeform-C and Freeform-E were prepared at equivalent drug concentrations. A 40 μL volume was injected into both the tumor center and periphery of each mouse using an insulin syringe. For gel groups, blue-light irradiation (395 nm) was applied at close range for 10 min to induce gelation, while this step was omitted for free-drug groups. Residual gel and surface materials were removed with 70% ethanol.

For the laser group, an 808 nm NIR source was applied at 75% output, targeting the tumor site for 5 min. For the radiotherapy (RT) group, irradiation was performed at 432, 2 cm, and 5 Gy. Tumor volume and body weight were monitored every three days using the same methods described above.

## 3. Results and Discussion

### 3.1. Hydrogel Characterization and Analysis

#### 3.1.1. Basic Properties

##### Chemical Structure Analysis

**Figure 1:**
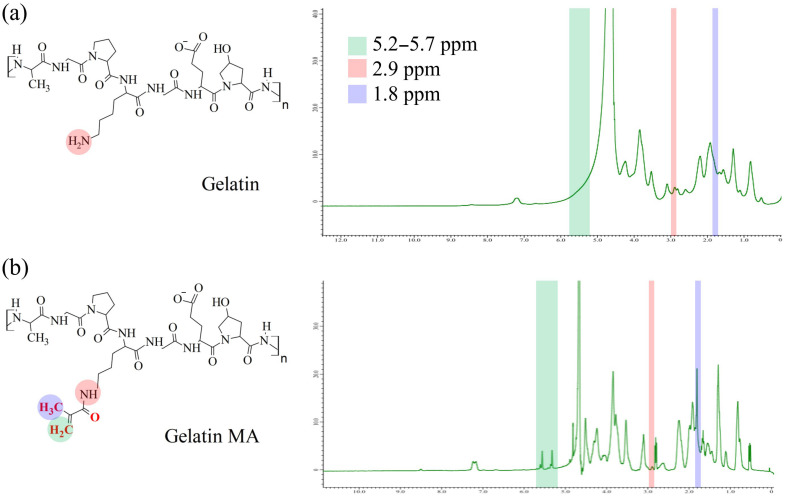
^1^H NMR spectra of (**a**) Gelatin and (**b**) Gelatin MA. The green-shaded region (5.2–5.7 ppm) corresponds to the vinyl proton signals of the methacryloyl group. The red-shaded region (2.9 ppm) corresponds to the amino proton signals of lysine. The blue-shaded region (1.8 ppm) corresponds to the methyl proton signals of the methacryloyl group.

Figure 1 presents the hydrogen-1 nuclear magnetic resonance (NMR) spectra of gelatin and Gelatin MA, both dissolved in deuterium oxide (D_2_O). Compared with gelatin, Gelatin MA exhibited a notably weaker absorption peak at 2.9 ppm, corresponding to the amine group protons of lysine residues [38]. This reduction is attributed to the dehydration reaction during methacryloyl substitution, indicating replacement of lysine amine protons. In addition, three peaks present in Gelatin MA but absent in gelatin were observed at 1.8, 5.2, and 5.7 ppm. The peak at 1.8 ppm corresponds to the methyl protons of the methacryloyl group, while the peaks at 5.2 and 5.7 ppm correspond to the vinyl group protons [38,39]. These spectral features—loss of lysine amine proton signals and appearance of methacryloyl methyl and vinyl proton signals—confirm the successful grafting of methacryloyl groups onto gelatin.

**Figure 2:**
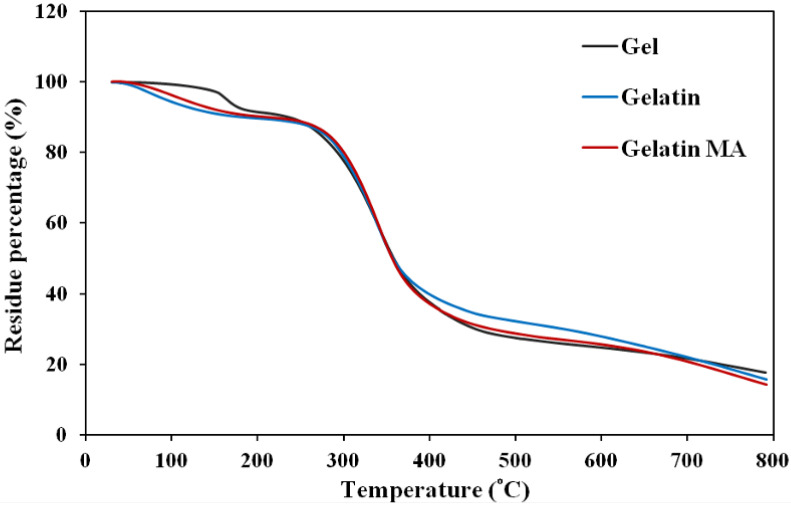
Thermogravimetric analysis (TGA) of Gel, Gelatin MA, and Gelatin. The curves show the weight changes in each material as the temperature increases.

Crosslinking typically increases polymer molecular weight and can influence thermal degradation behavior. To evaluate these effects in the hydrogel, TGA was performed on freeze-dried gelatin, Gelatin MA powders, and dried hydrogel samples, monitoring the extent of weight loss as temperature increased. The derivative weight curves (DTG) were then plotted, where the peak positions indicate the corresponding degradation temperatures. As shown in Figure 2 and Table 2, all three materials exhibited a primary degradation temperature between 338.4 and 341.7 °C, corresponding to gelatin backbone degradation. Gelatin and Gelatin MA showed minor weight losses at 71.6 °C and 102.7 °C, respectively, whereas the hydrogel remained stable below 100 °C. This indicates that the hydrogel is thermally stable under the temperature conditions required for aqueous and enzymatic reactions. The hydrogel exhibited a distinct degradation event at 163.4 °C, consistent with the removal of bound or hydrated water, as thermal events below 180 °C are typically associated with dehydration of proteins and polysaccharides.

Previous work by Klein et al. on genipin-crosslinked chitosan reported that weight losses between 100 °C and 270 °C are attributable to covalent bond cleavage leading to polymer structural weakening, while differences in weight loss below 100 °C reflect variations in hydrophilicity, with greater dehydration weight loss indicating higher hydrophilicity of the material [40]. In the present study, all samples were dried prior to TGA; thus, the observed differences in dehydration temperatures can be attributed to the hydrogel’s covalently crosslinked network, which restricts the release of bound water within molecular segments, requiring higher temperatures for dehydration. The degradation of the hydrogel at 163.4 °C was likely due to covalent bond cleavage within the crosslinked structure, resulting in reduced molecular weight or monomer formation, after which the thermal degradation profile resembled that of gelatin and Gelatin MA. Overall, the TGA results indicate that the hydrogel was highly stable below 100 °C, with notable weight loss between 100 and 200 °C arising from intermolecular dehydration and cleavage of crosslink-generated covalent bonds. Apart from this, the hydrogel and Gelatin MA exhibited thermal degradation trends similar to gelatin, suggesting that methacryloyl modification and gelation do not significantly alter the primary gelatin structure.

##### Gelation Test

**Figure 3:**
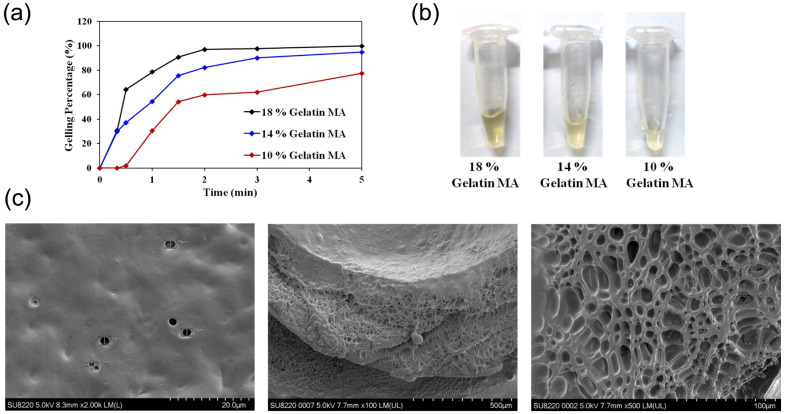
Gelling kinetics and microstructural characterization of Gelatin MA hydrogels at varying concentrations. (**a**) Gelation profiles of 10%, 14%, and 18% Gelatin MA exposed to blue light, showing gelation ratio as a function of exposure time. (**b**) Corresponding photographs depict the gel volumes of each concentration after 2 min of blue-light irradiation. (**c**) SEM images of the hydrogel surface (**left**, scale bar = 20 µm), cross-section (**middle**, scale bar = 500 µm) and zoomed-in detailed structure of gel (**right**, scale bar = 100 µm).

For a hydrogel-based drug delivery system to be suitable for clinical application, it should possess rapid gelation properties. This minimizes the risk of dilution by body fluids due to prolonged gelation time or undesired gelation at non-target sites, which could lead to unintended drug release. To evaluate the gelation time of this hydrogel system and to optimize the gelation conditions, gelation tests were performed. Gelatin MA at different concentrations was exposed to a blue-light source of fixed power for various time intervals. At each time point, the un-gelled Gelatin MA solution was removed, and the weight of the remaining gel was measured. The gelation percentage was calculated as the ratio of gel weight to the total weight of the Gelatin MA–photosensitizer mixture. At a concentration of 18% (*w*/*w*), Gelatin MA achieved a gelation ratio of over 95% within 2 min (Figure 3a), and even after drug incorporation, gelation occurred within 5 min (Figure 3b). The image of Gel MA before and after gelation is as demonstrated in Figure S1. These results indicate that this hydrogel system exhibits rapid gelation and is easy to handle, requiring only the avoidance of direct fluorescent light exposure after mixing Gelatin MA with the photosensitizer to prevent premature polymerization.

As shown in Figure 3c, freeze-dried hydrogels were sectioned, and their surface and cross-sectional morphologies were examined by scanning electron microscopy (SEM). Post-gelation, Gelatin MA exhibited a smooth surface and a porous internal structure. Smooth hydrogel surfaces are generally thought to interact favorably with cells and the extracellular matrix, enhancing adhesion, while hydrogels formed through crosslinking typically possess porous internal architectures. This porous morphology is consistent with previous Gelatin MA studies [41]. When hydrogels are used as scaffolds for cell culture or wound closure, such porosity facilitates cell attachment and extracellular matrix secretion [42]. SEM imaging of the hydrogel cross-section revealed pore sizes ranging from 100 to 300 µm, with most pores being smaller than 200 µm. Since hydrogels intended for three-dimensional cell culture scaffolds require pores larger than 200 µm to enable cell migration and proliferation within the structure [43], the pore size distribution in this Gelatin MA hydrogel is not optimal for promoting extensive cell growth inside the hydrogel.

#### 3.1.2. Photothermal Material Analysis

##### Physicochemical Characterization

**Figure 4:**
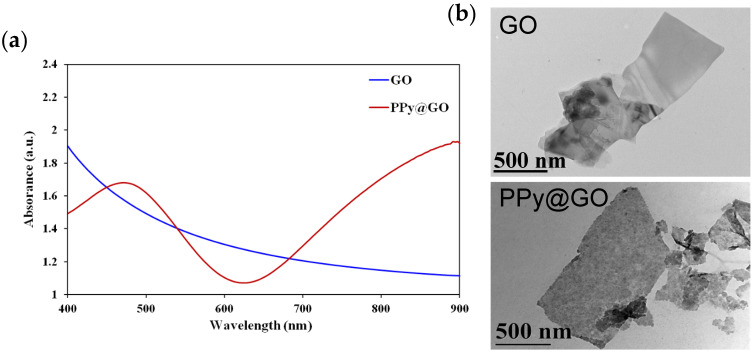
Optical and morphological characterization of GO and PPy@GO. (**a**) UV–Vis absorption spectra of GO and PPy@GO. (**b**) Transmission electron microscopy (TEM) images showing sheet-like morphology (top: GO; bottom: PPy@GO). Scale bars: 500 nm.

During the synthesis of PPy@GO, the addition of catalyst and pyrrole to the initially brown-black GO solution resulted in a distinct color change to deep black. Although this visible change suggested successful modification, further analyses were necessary to confirm PPy@GO formation. Therefore, both PPy@GO and GO were characterized using UV–Vis absorption spectroscopy. As shown in Figure 4a, the absorption spectrum of GO exhibited a smooth, gradual decrease in absorbance from 400 nm toward longer wavelengths. In contrast, PPy@GO displayed an absorption band at 480 nm and a gradual increase in absorbance beyond 600 nm, forming a smooth upward slope—features absent in GO (Figure 4a). These spectral characteristics are consistent with previously reported polypyrrole absorption profiles, confirming the successful synthesis of PPy@GO.

Transmission electron microscopy (TEM) images in Figure 4b revealed that both PPy@GO and GO retained thin sheet-like morphologies, either as single layers or multilayer stacks. In PPy@GO, darker spots were observed dispersed on the GO sheets, corresponding to polypyrrole particles attached to the GO surface. Both PPy@GO and GO exhibited lateral dimensions of 200–700 nm. According to Liu et al., GO within this size range is primarily metabolized by the reticuloendothelial system (RES) via the lungs and liver, with minimal distribution to other organs [25]. Compared to GO with lateral sizes above 2 µm, which tends to partially accumulate in the lungs, sub-2 µm GO shows a dynamic distribution between the liver and lungs. Specifically, lung GO concentrations decrease to very low levels—comparable to those in the spleen and kidneys—within 3 h post-administration, while liver GO concentration peaks at 5–10 min and then declines gradually. These findings indicate that small-sized GO can be efficiently captured by the liver and rapidly metabolized, resulting in higher total body clearance and a shorter plasma half-life. Therefore, in this study, polypyrrole was synthesized on GO sheets with lateral sizes of 200–700 nm. As a photothermal material, PPy@GO in this size range is expected to undergo rapid hepatic metabolism, exhibit low long-term biodistribution, and offer improved in vivo safety.

##### In Vitro Photothermal Performance Test

To investigate whether the photothermal material could absorb near-infrared (NIR) laser irradiation and exhibit a photothermal effect in vitro, two groups—P-gel and Gel—were tested. For each group, 100 μL of pre-gelled sample was prepared in microcentrifuge tubes. The samples were irradiated with an 808 nm NIR laser at 75% output power for 3 min, and temperature changes were recorded using a far-infrared (thermal) camera.

**Figure 5:**
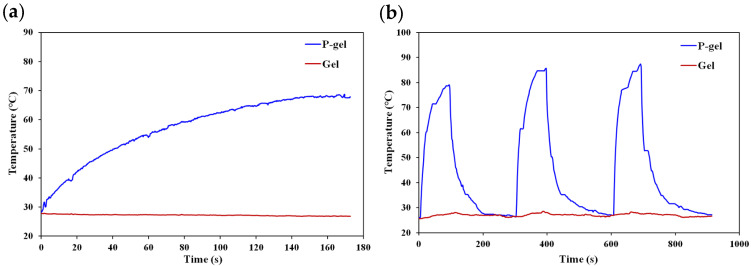
In vitro photothermal effect of P-gel and Gel. (**a**) Single-cycle temperature rise during 3 min of 808 nm near-infrared laser irradiation (75% output power). (**b**) Repeated irradiation test, with each cycle consisting of 90 s irradiation followed by cooling to room temperature, to evaluate photothermal stability.

As shown in Figure 5a, the P-gel group reached 50 °C within 1 min and 68 °C after 3 min of irradiation. In contrast, the Gel control group exhibited temperature fluctuations within ±1 °C, indicating negligible temperature change. These results demonstrate that the P-gel used in this study exhibited a distinct photothermal effect under identical NIR irradiation parameters, whereas hydrogels without PPy@GO showed no temperature rise, confirming that PPy@GO loading is essential for the photothermal response.

In the repeated heating test shown in Figure 5b, 100 μL of pre-gelled P-gel or Gel was adhered to glass slides at room temperature. Samples were irradiated with an 808 nm NIR laser at 75% output power for 90 s, then allowed to cool to room temperature before the next irradiation cycle. For P-gel, the maximum temperatures recorded in three successive irradiations were 79 °C, 85.6 °C, and 87.3 °C, corresponding to temperature increases of 52.9–61.2 °C. Notably, the peak temperature did not decrease with repeated cycles; instead, it showed a slight upward trend. In contrast, Gel under the same irradiation conditions reached a maximum of 28.6 °C, with temperature increases of only 1.3–2.4 °C. These results confirm that P-gel can withstand multiple heating cycles without loss of photothermal performance, indicating not only a significant photothermal effect, but also excellent thermal stability of the PPy@GO-containing material.

##### Cell Cytotoxicity

In addition to assessing the temperature rise in P-gel induced by its photothermal effect, it was necessary to evaluate whether the magnitude of this temperature change in vitro was sufficient to affect cell viability. For this purpose, the following six experimental groups were prepared: Gel and P-gel samples adhered to the surface of cell culture plates, and a control group without any material, each further divided into irradiated and non-irradiated subgroups. For irradiated groups, samples were exposed to an 808 nm NIR laser at 75% output power for 10 min. For each material type, the cell viability under NIR irradiation was calculated as the XTT assay absorbance value of the irradiated subgroup divided by that of the corresponding non-irradiated subgroup, expressed as a percentage, to evaluate the effect of the photothermal response on cell survival.

**Figure 6:**
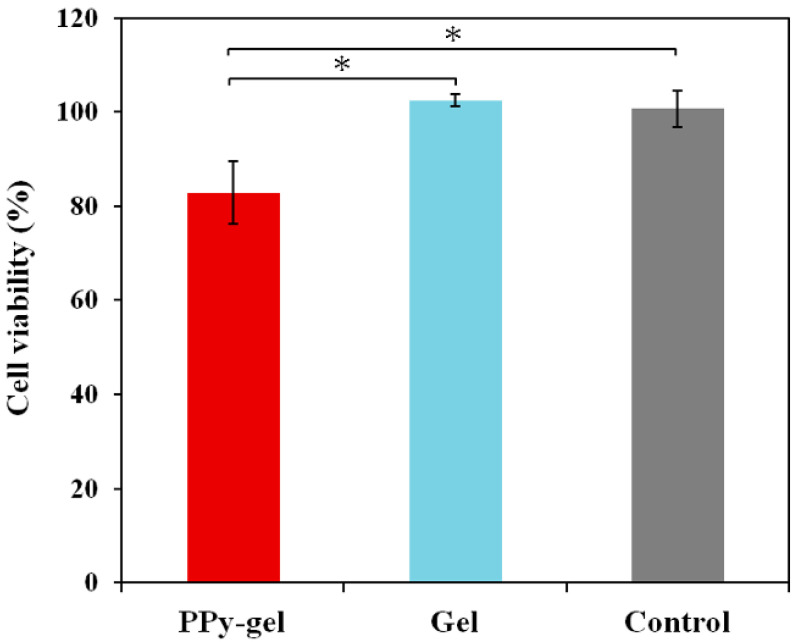
Cytotoxicity of control (100.7%), Gel (102.4%), and P-gel (85.3%) groups after 10 min irradiation with an 808 nm near-infrared laser (75% output power). Cell viability was measured 24 h post-irradiation using the XTT assay. *t*-test: * represent statistical significance (*p* < 0.05).

As shown in Figure 6, the cell viability at 24 h after 808 nm NIR laser irradiation was 85.3% for the P-gel group, 102.4% for the Gel group, and 100.7% for the control group. Statistical analysis (*t*-test) showed that the P-gel group exhibited a significant difference compared to both the Gel group and the control group (*p* < 0.05). Specifically, 808 nm NIR irradiation did not exert cytotoxicity toward the control or Gel groups, and their similar cell viability values further confirm the excellent biocompatibility and low cytotoxicity of the hydrogel itself. In contrast, the PPy@GO-loaded hydrogel produced heat upon NIR irradiation, and the temperature rise was sufficient to induce cytotoxicity. The photothermally induced cytotoxic effect in this drug delivery system was only observed when the hydrogel contained PPy@GO and was subjected to 808 nm NIR laser irradiation.

### 3.2. Drug Release and Cytotoxicity of Drug-Loaded Hydrogels

#### 3.2.1. Drug Release Profile

An effective hydrogel-based drug delivery system should be capable of sustained drug release; therefore, evaluation of drug release behavior under physiological conditions is essential. In drug release experiments, pH 7.4 PBS buffer is commonly used to simulate the neutral pH of normal body fluids in healthy tissues, whereas pH 6 is used to mimic the mildly acidic environment of tumors. In this study, collagenase—a matrix metalloproteinase secreted by cancer cells to degrade the extracellular matrix and facilitate proliferation and invasion—was also introduced at a concentration of 500 ng/mL to more closely simulate the tumor microenvironment. E-gel was used as the model system to investigate EPI release, with the following three experimental groups: pH 6 + collagenase, pH 6, and pH 7.4.

**Figure 7:**
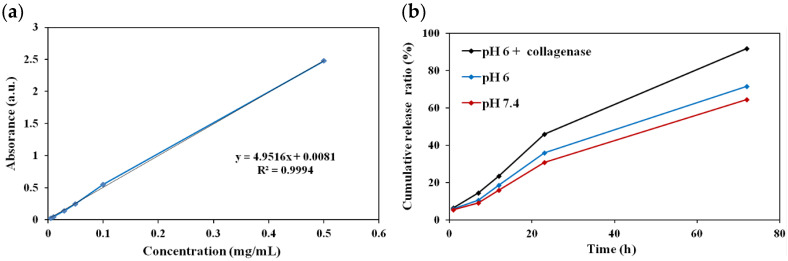
EPI calibration and pH/collagenase-responsive release from E-gel. (**a**) Calibration curve of EPI concentration based on UV–Vis absorption spectra. (**b**) Cumulative EPI release from E-gel under simulated tumor microenvironments (black lines: pH 6.0 + 500 ng/mL collagenase, blue lines: pH 6.0) and a simulated normal physiological environment (red line: pH 7.4).

As shown in Figure 7, in the neutral pH 7.4 PBS environment, drug release was very slow: E-gel released only 15.8% of EPI within 12 h and 64.4% after 72 h. Under acidic conditions (pH 6), 18.6% of EPI was released within 12 h and 71.5% after 72 h. In the collagenase-containing group, 14.5% of EPI was released within just 3 h, 23.4% within 12 h, and 91.7% after 72 h. Compared with pH 7.4, the acidic condition led to greater drug release. This difference arose because, in acidic environments, the amino groups on the gelatin chains are more readily protonated to form positively charged ammonium groups, which repel one another, as well as protons in the solution, due to their like charges. This electrostatic repulsion loosens the hydrogel network, allowing EPI to diffuse out more rapidly. In the pH 6 + collagenase condition, degradation of RGD sequences in gelatin residues by collagenase further disrupted the hydrogel structure, resulting in the highest drug release.

Since Gelatin MA can be degraded by collagenase, it is a substrate for this enzyme, and its RGD sequence—which confers cell- and tissue-adhesive properties—retains bioactivity in the modified form. Thus, this hydrogel drug delivery system demonstrates sustained drug release, with selectively higher release under acidic and collagenase-containing conditions compared to neutral pH, representing a stimulus-responsive drug release behavior.

#### 3.2.2. Cytotoxicity Assay

To evaluate the intrinsic cytotoxicity and predict the biocompatibility of the hydrogel material itself, as well as that of hydrogels loaded with photothermal agents and drugs, cytotoxicity tests were performed. Experimental groups were classified according to the presence or absence of photothermal material and drugs, and by drug formulation type. The eight groups included the following: Gel, P-gel, Cisplatin-gel (C-gel), Epirubicin-gel (E-gel), Cisplatin–Epirubicin-gel (CE-gel), Freeform-Cisplatin (Freeform-C), Freeform-Epirubicin (Freeform-E), and Freeform-Cisplatin–Epirubicin (Freeform-CE). The following two cell models were used: the human glioblastoma cell line U87-MGFL and the murine brain tumor cell line MBR-614. Cell viability was measured at 12, 24, 48, and 72 h after exposure to the respective materials or drug formulations. All control groups contained no materials. To avoid the cells being covered by the gel, which would inhibit cell growth and cause a non-therapeutic effect interfering with cell viability, we added hydrogels to the wells before the cells.

**Figure 8:**
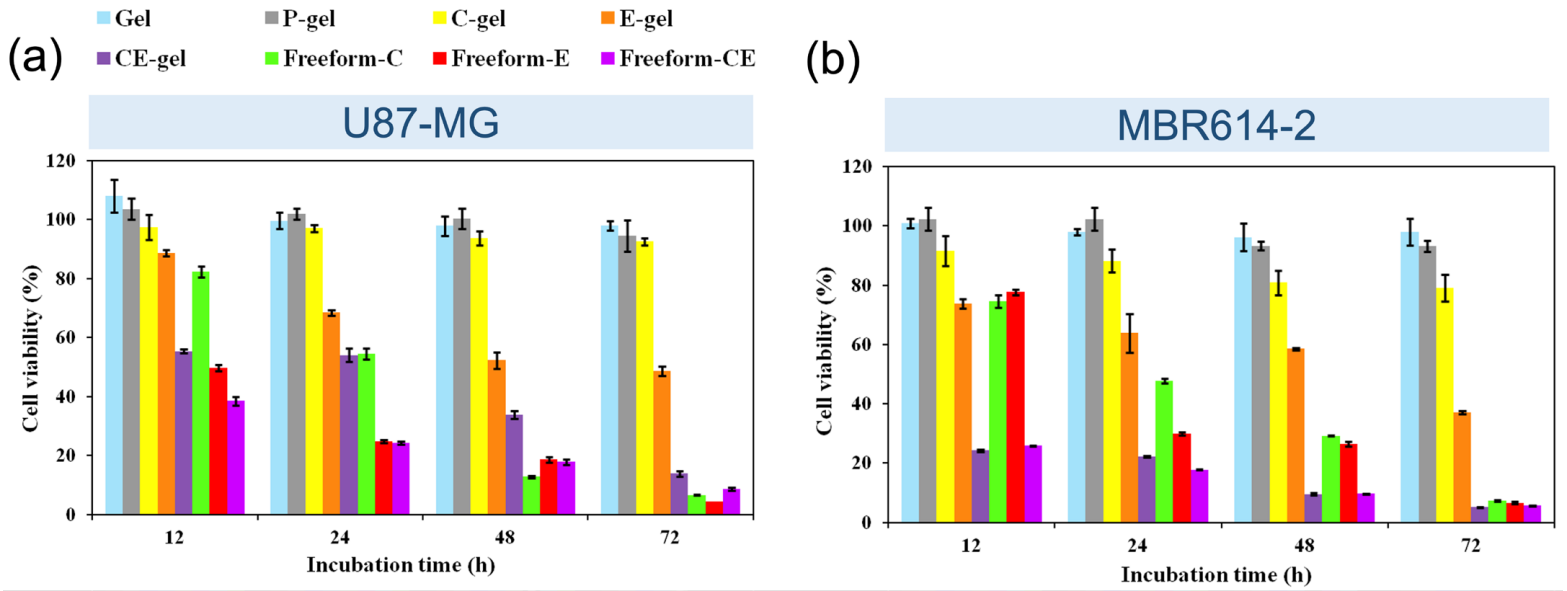
Cytotoxicity and EPI uptake in U87-MGFL and MBR-614 cells after co-culture with various hydrogel and free-drug formulations. (**a**) Cell viability of U87-MGFL cells after 12, 24, 48, and 72 h co-culture with Gel, P-gel, C-gel, E-gel, CE-gel, Freeform-C, Freeform-E, and Freeform-CE. (**b**) Cell viability of MBR-614 cells under the same conditions.

In experiments using U87-MGFL cells (Figure 8a), cell viability for the Gel group ranged from 97.9% to 107.9% across the four time points, while the P-gel group ranged from 94.5% to 103.6%, indicating negligible cytotoxicity for both groups. For single-drug hydrogel formulations, E-gel viability decreased from 88.6% at 12 h to 48.6% at 72 h, while C-gel ranged from 97.4% to 92.5%. In comparison, Freeform-E viability dropped sharply from 49.8% to 4.4% and Freeform-C from 82.3% to 6.6% over the same period. CE-gel showed a viability decreasing from 55.4% to 13.8%, whereas Freeform-CE declined from 38.5% to 8.5%. Statistical analysis showed no significant difference between Gel and P-gel at any time point (*p* = 0.0725–0.1267). However, for C-gel, E-gel, and CE-gel, their viabilities were significantly different from their respective free-drug counterparts at all time points (*p* < 0.05). These results indicate that both the hydrogel material and the photothermal material exhibited very low cytotoxicity, while hydrogel-loaded drugs retained cytotoxic effects but at a reduced severity due to their slower release compared to free drugs. Furthermore, the combination of cisplatin and EPI in CE-gel exhibited greater cytotoxicity than either drug alone, confirming the synergistic effect described earlier.

For MBR-614 cells (Figure 8b), Gel viability ranged from 96.1% to 100.9% and P-gel from 93.1% to 102.4%. Among the single-drug hydrogel groups, C-gel viability ranged from 91.5% to 79.1% and E-gel from 73.8% to 37.2%. Freeform-C dropped from 74.6% to 7.4% and Freeform-E from 77.7% to 6.6%. CE-gel ranged from 24.2% to 5.1%, while Freeform-CE ranged from 25.8% to 5.7%. The cytotoxicity trends for MBR-614 cells were similar to those observed in U87-MGFL cells, with hydrogel-loaded drug formulations showing lower cytotoxicity than free drugs and the cisplatin–EPI combination effectively inhibiting cell growth. Notably, hydrogel degradation was observed in the MBR-614 groups during the experiments, which may explain the heightened sensitivity of this cell line to drug-loaded hydrogels.

Overall, results from both cell lines indicate that Gel and P-gel (without irradiation) maintained high cell viability, demonstrating low cytotoxicity and high biocompatibility. The ability of the hydrogel to preserve drug activity while enabling sustained release resulted in detectable cytotoxicity from hydrogel-loaded drugs, but at a slower and less severe rate than free-drug formulations. Single-drug-loaded hydrogels showed lower cytotoxicity than dual-drug formulations, supporting the enhanced effect of drug combination in this hydrogel system. The gradual increase in cytotoxicity over time for hydrogel-loaded groups further confirms that the observed effects were due to the slow, sustained release of drugs from the hydrogel and subsequent cellular uptake.

#### 3.2.3. Cellular Fluorescence Imaging

Previous cytotoxicity and drug release studies demonstrated that drug-loaded hydrogels can gradually release their payloads and induce cytotoxicity. However, the shortest time point assessed in the cytotoxicity assays was 12 h. The detailed temporal pattern of drug release from hydrogels and subsequent cellular uptake within shorter intervals remains to be elucidated. To address this, U87-MGFL and MBR-614 cells were seeded in 6-well culture plates, and 40 μL of E-gel was added to each well. Co-cultures were maintained for 1, 3, 5, 7, or 12 h. Following incubation, E-gel samples were removed, the culture medium was discarded, and cells were washed. Control groups received 40 μL of Freeform-E solution at the same concentration and were co-cultured for 1 h before removal and washing. All cells were then stained with Hoechst 33342 (blue fluorescence) to visualize nuclei. EPI fluorescence (orange-red) was recorded, and overlap between EPI and Hoechst signals was examined to assess nuclear drug localization.

**Figure 9:**
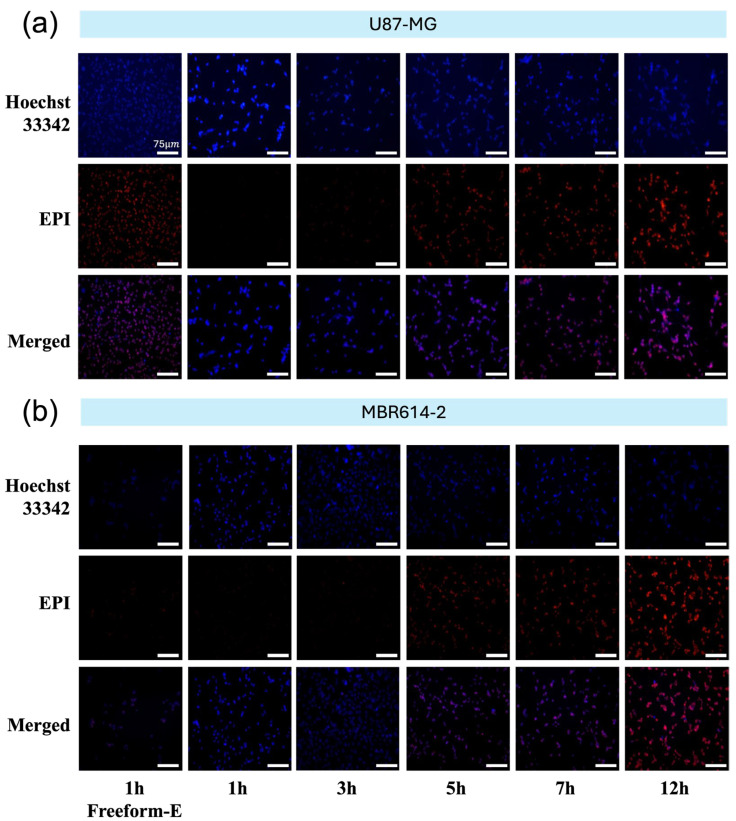
Cellular fluorescence images of (**a**) U87-MGFL and (**b**) MBR-614 cells co-cultured with E-gel or free-form EPI solution for 1, 3, 5, 7, and 12 h. Nuclei were stained with Hoechst 33342 (**top**, blue), EPI fluorescence is shown in red (**middle**), and merged images are shown at the **bottom**. Scale bar = 75 μm.

Imaging conditions, including software exposure time and enhancement settings, were kept constant across all samples. As shown in Figure 9, EPI fluorescence intensity within cells increased with a longer E-gel co-culture time. From approximately 5 h onward, distinct EPI fluorescence was visible within cells under fixed exposure conditions. By 12 h, Hoechst 33342 staining revealed morphological changes—fluorescence spread throughout the cytoplasm—indicating nuclear DNA redistribution due to apoptosis. At this stage, cell numbers declined, and cellular aggregation was observed. In the Freeform-E control group, strong EPI fluorescence was detected after only 1 h, under identical exposure conditions. Hoechst 33342 staining also indicated nuclear morphological changes similar to those in the 12 h E-gel group. For both E-gel and Freeform-E, merged fluorescence images showed overlapping EPI and Hoechst signals within nuclei, indicating that EPI released from E-gel entered the nucleus to exert cytotoxic effects. These fluorescence imaging results confirm that drugs encapsulated within the hydrogel retained their bioactivity and that the hydrogel delivery system enabled gradual drug release, cellular uptake, and nuclear localization.

### 3.3. Animal Experiments Results

#### 3.3.1. In Vivo Gelation Test

To evaluate whether Gelatin MA could undergo gelation within the complex physiological environment of a living organism and to observe the morphology of the resulting gel, the hydrogel was labeled with the fluorescent dye FITC (excitation wavelength: blue light). FITC-labeled Gelatin MA was injected subcutaneously into mice, which were divided into two groups based on the presence or absence of blue-light irradiation.

**Figure 10:**
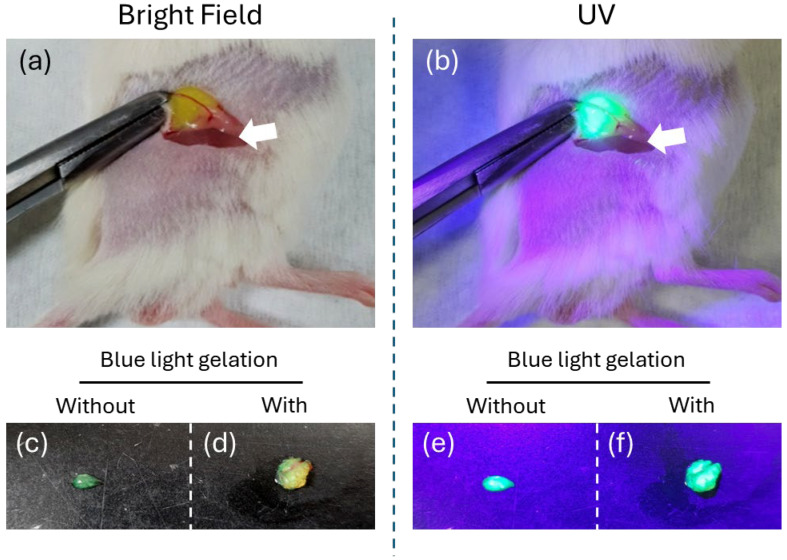
Image of dissected tissue showing FITC-gel formation from in vivo gelation test using FITC-labeled hydrogel visualized under blue-light excitation. (**a**,**b**) show morphology of FITC-gel under bright field (**a**) morphology of FITC-gel under UV light (**b**). (**c**,**d**) show extracted FITC-gel ex vivo tissue without (**c**) and with (**d**) blue light excitation under bright field. The corresponding images under UV light are shown in (**e**,**f**). White arrow indicates the tissue surrounding the gel.

As shown in Figure S3a, the blue-light-irradiated group displayed a clear bulge on the ventral side of the mouse, and images captured under blue-light excitation (Figure S3b) show green FITC fluorescence at the bulging site. As shown in Figure S3c, the non-irradiated group exhibited a less pronounced and flatter bulge; upon dissection (Figure S3d), the injected area contained liquid FITC–Gelatin MA mixture, indicating the absence of gel formation. As shown in Figure 10a, dissection of tissue from the irradiated group revealed a solid mass separated from the surrounding tissue; under blue-light excitation, as shown in Figure 10b, this mass exhibited green fluorescence, while the surrounding tissue showed no fluorescence, confirming it as FITC-gel formed in vivo. The Figure 10d,f show FITC-gel removed from the animal, maintaining its three-dimensional shape ex vivo, demonstrating its robust structure and good mechanical properties while the gel without blue light gelation remains liquid form (Figure 10c,e).

This in vivo gelation experiment confirms that Gelatin MA can overcome the challenges of a complex, fluid-containing physiological environment to form a gel in vivo. The resulting gel demonstrated strong structural integrity, while no gel formation occurred in the non-irradiated control group, indicating that gelation requires blue-light activation. This specificity reduces the risk of unintended gelation prior to clinical application.

#### 3.3.2. In Vivo Study of Photothermal Effect

In addition to physicochemical analyses of PPy@GO and in vitro verification of the photothermal effect of P-gel, it was necessary to investigate the performance of the photothermal effect in living animals to confirm whether the temperature rise induced by P-gel is sufficient for potential future clinical applications in hyperthermia therapy. In this experiment, subcutaneously injected Gel served as the control group, while P-gel was the experimental group. Equal volumes of P-gel or Gel were implanted subcutaneously into C57BL/6 mice. The implanted sites were then irradiated with an 808 nm near-infrared (NIR) laser for 5 min, and temperature changes were recorded both numerically and through thermal infrared imaging. The thermal images before and after irradiation were compared to determine the temperature differences in the measured regions.

**Figure 11:**
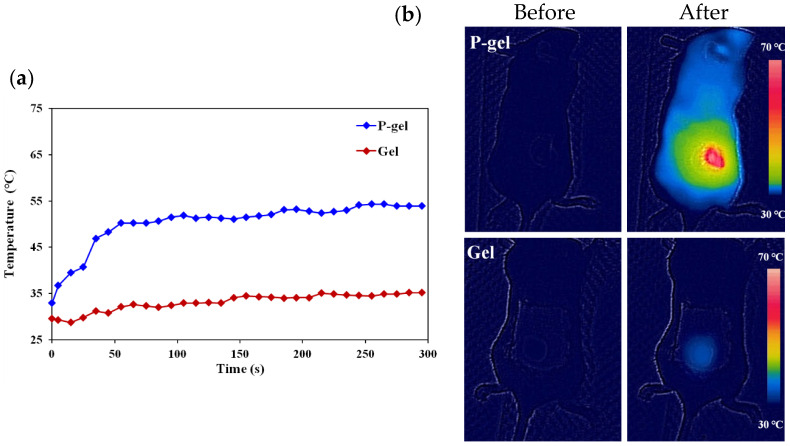
In vivo photothermal effect study. (**a**) Temperature variation curves of Gel and P-gel after subcutaneous gelation and 5 min irradiation with an 808 nm near-infrared laser. (**b**) Infrared thermal images of Gel and P-gel before (**left**) and after (**right**) 5 min of 808 nm near-infrared laser irradiation.

The in vivo photothermal effect study was performed to evaluate the if the PPy@GO-loaded gel could generate enough photothermal effect during applications. The result is as shown in Figure 11a and the temperature change is as shown in Figure S2. In the Gel group, after 5 min of 808 nm NIR irradiation, the measured site increased from 30.7 °C to 33.5 °C, a rise of only 2.8 °C, which was insufficient to induce apoptosis, as the final temperature (33.5 °C) remained below the threshold for cytotoxicity. In contrast, the P-gel group showed a temperature increase from 32.9 °C to 53.0 °C, an elevation of 20.1 °C. The temperature was maintained above 52 °C for 190 s and above 53 °C for 115 s. According to the study by Dewhirst et al. on thermal injury in human skin, exposure to 53 °C for 90 s is sufficient to induce cell necrosis and cause tissue necrosis. Therefore, the temperature rise and duration achieved in this study are adequate to induce apoptosis and necrosis, consistent with the results of previous in vitro experiments. Thermal infrared images in Figure 11b also clearly demonstrate temperature changes in mice before and after NIR irradiation. These results indicate that the photothermal effect of P-gel can generate heat in vivo that increases the temperature toward 50 °C, and that the magnitude of this temperature elevation is sufficient to cause damage to cancer cells.

#### 3.3.3. Antitumor Efficacy of Hydrogels Loaded with Drugs and Photothermal Material

The goal of this study was to evaluate the potential clinical applicability of the hydrogel drug delivery system. Beyond physicochemical characterization, stability testing, drug release, and cytotoxicity studies, it was necessary to assess its in vivo antitumor efficacy using animal models. In this experiment, C57BL/6 mice were subcutaneously implanted with U87-MGFL cells to establish tumor models. A 40 μL volume of hydrogel—with or without drugs and photothermal materials—was injected into and around the tumors. Gelation was induced in situ with blue-light irradiation. For free-drug controls, equivalent doses of cisplatin and/or epirubicin were administered in solution form via injection. For groups requiring additional therapy, tumors were exposed to an 808 nm NIR laser (5 min) or subjected to radiotherapy (RT). The following seven groups were evaluated: control, P-gel, CEP-gel, CEP-gel + RT, CEP-gel + RT + Laser, intravenous cisplatin + RT (IV. C + RT), and intravenous epirubicin (IV. E). Tumor volume and body weight were measured every three days, and overall survival was recorded.

**Figure 12:**
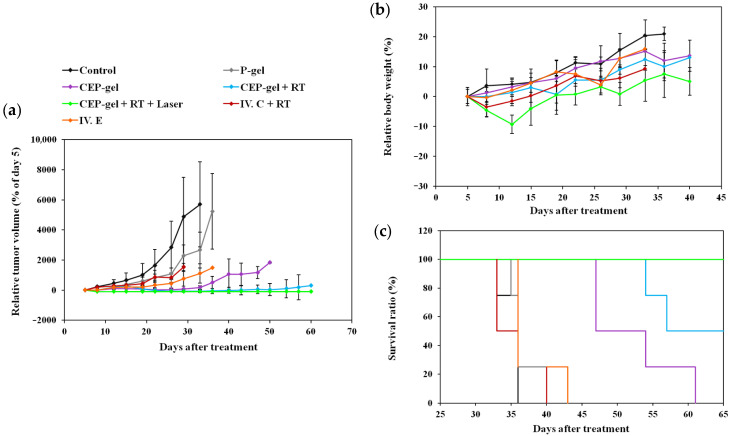
In vivo antitumor efficacy of the hydrogel drug delivery system. Tumor-bearing mice were treated with intratumoral injection of P-gel, CEP-gel, CEP-gel + RT, CEP-gel + RT + Laser, or with intravenous cisplatin + RT or epirubicin. Shown are (**a**) relative tumor volume compared with baseline, (**b**) relative body weight compared with baseline, and (**c**) survival rate (*n* = 4 per group).

Tumor volume served as the primary indicator of antitumor efficacy. As shown in Figure 12a, tumor volumes in the P-gel and IV. E groups increased rapidly. The IV. C + RT and CEP-gel groups showed moderate inhibition, while CEP-gel + RT was more effective, and CEP-gel + RT + Laser resulted in complete tumor regression by day 8. By day 36, the tumor volumes in the control and P-gel groups reached 2007 mm^3^ and 1996 mm^3^, respectively, requiring sacrifice. The IV. E group exhibited a similar tumor growth trend, but mice died or became moribund before tumors reached the endpoint. The IV. C + RT and CEP-gel groups suppressed tumor growth for approximately 30 days, after which tumor regrowth surpassed humane endpoints on days 43 and 60, respectively. The CEP-gel + RT group suppressed growth for more than 50 days, with a residual tumor volume of 197 mm^3^ at day 65. Strikingly, CEP-gel + RT + Laser caused tumor shrinkage and complete disappearance after day 8. These results indicate that P-gel was biocompatible and did not inhibit tumor growth, while the free-drug groups (IV. E and IV. C + RT) exhibited limited efficacy due to short half-life and systemic distribution. CEP-gel released cisplatin and epirubicin gradually, sustaining tumor suppression for around 30 days. CEP-gel + RT benefited from both slow drug release and radiosensitization, prolonging suppression beyond 54 days. Most notably, CEP-gel + RT + Laser achieved the most pronounced effect, combining chemotherapy, radiotherapy, and hyperthermia to eradicate tumors.

Body weight was monitored as an indicator of systemic toxicity (Figure 12b). All groups showed a gradual weight increase without significant intergroup differences, suggesting minimal or negligible side effects. The P-gel group reconfirmed its low cytotoxicity. In the IV. C + RT group, a transient weight decrease was observed at treatment initiation, likely attributable to both cisplatin toxicity and overlap between the irradiation field and abdominal cavity, which may have affected digestion. No significant weight loss was observed in the IV. E group, indicating mild or absent epirubicin toxicity at this dosage.

Survival served as an additional index of therapeutic efficacy (Figure 12c). Median overall survival days was as follows: control, 35; IV. E, 33; P-gel, 36; IV. C + RT, 36; CEP-gel, 47; CEP-gel + RT, 57. In the CEP-gel + RT + Laser group, no mice died or reached humane endpoints by day 65 (study endpoint), with survival exceeding 50%. Compared with the control, CEP-gel extended median survival by 12 days, and CEP-gel + RT and CEP-gel + RT + Laser extended survival by at least 22 days.

These in vivo findings confirm that while free-form drugs and simple P-gel lacked durable efficacy, the CEP-gel hydrogel system provided sustained drug release, enhanced tumor inhibition, and significant survival benefit. The addition of RT and especially RT combined with hyperthermia yielded synergistic effects, with CEP-gel + RT + Laser producing complete tumor regression and the best antitumor outcome.

In this study, neither the hydrogel material itself nor the administered drug dosages caused significant side effects such as weight loss in mice. However, analysis of tumor volume changes revealed that Gelatin MA and PPy@GO, due to their high biocompatibility, did not interfere with cancer cell growth and, thus, allowed tumors to continue expanding. Free-form drug administration exhibited limited antitumor efficacy, constrained by dosage and systemic distribution. In contrast, drug-loaded hydrogels effectively suppressed tumor growth by gradually releasing drugs, and when combined with photothermal and radiotherapy, demonstrated superior therapeutic outcomes, including tumor regression and prolonged survival. These preliminary animal results confirm that the hydrogel drug delivery system, when integrated with photothermal therapy and radiotherapy, can effectively inhibit tumor growth and extend survival in tumor-bearing mice. Importantly, this system achieved antitumor efficacy without causing significant side effects, attributable to the hydrogel’s ability to preserve the stability and bioactivity of drugs and photothermal agents, sustain drug release, and maintain high local drug concentrations in the tumor region over an extended period.

## 4. Discussion

Drug delivery remains a critical challenge in cancer therapy. Numerous carriers, such as nanoparticles, liposomes, and hydrogels, have been investigated. Hydrogels are particularly attractive owing to their ability to achieve localized and sustained drug release, ease of application, and capacity to fill irregular postoperative cavities. These features are especially advantageous in brain tumors, where direct application enables prolonged local drug retention while potentially overcoming the blood–brain and blood–tumor barriers.

An effective hydrogel system for cancer therapy must meet several requirements, as follows: use of readily available and low-toxicity materials; preservation of drug stability; controlled and sustained release; and rapid in situ gelation after administration. The traditional method of hydrogel formulation is making a dried gel and implanting the drug-containing gel into the target site. However, the brain is fragile and irregularly shaped. Therefore, in situ gelation in the brain is a better way compared to making a gel and then performing implantation. In this study, methacrylated gelatin (Gelatin MA) was employed as the hydrogel matrix, crosslinked via blue-light initiation in the presence of VA-086. The platform was designed to co-deliver cisplatin and epirubicin for chemo- and radiosensitization, while incorporating PPy@GO to enable photothermal therapy, thus achieving multimodal treatment within a single implantable system.

Gelatin MA represents a relatively recent biomaterial with extensive use in tissue engineering, owing to its biocompatibility, low cytotoxicity, and moderate mechanical strength. Consistent with previous reports, ^1^H NMR confirmed successful methacrylation while preserving the majority of bioactive peptide residues responsible for adhesion and biodegradation [44]. Vinyl proton signals at ~5.2 and 5.7 ppm, together with a methyl signal at ~1.8 ppm, confirmed introduction of methacryloyl groups, while the reduction in lysine amine proton signals supported substitution at lysine residues. These features confirm successful methacrylation; apart from introducing methacryloyl functionality, the chemical structure of gelatin remained largely unchanged, indicating that Gelatin MA retains gelatin’s adhesion and biodegradation properties.

Evaluation of gelation dynamics showed that an 18 wt% Gelatin MA formulation achieved >95% gelation within 2 min, with drug-loaded gels forming within 5 min. Rapid gelation is clinically favorable, minimizing diffusion of unpolymerized material and reducing off-target drug spread. SEM analysis revealed smooth surfaces with porous internal morphology. Pore sizes below 200 μm, while less suitable for cellular ingrowth, are well suited for localized depot functions. This indicates that the sponge-like structure in the dried gel SEM image brings benefits for therapeutic agents’ loading and controlled release. Thermogravimetric analysis revealed a primary decomposition temperature of 338–342 °C in gelatin, Gelatin MA, and the crosslinked gels, consistent with degradation of the gelatin backbone. In addition, crosslinked gels exhibited a minor degradation event at ~163.4 °C, absent in unmodified gelatin, corresponding to dehydration of absorbed water and cleavage of covalent bonds formed during crosslinking. Although only ~5% of functional groups participated in the reaction, the presence of this event provides clear evidence for covalent bond formation and gel network establishment.

Drug release studies demonstrated slow and sustained release of epirubicin, with enhanced release under mildly acidic conditions and marked acceleration in the presence of collagenase. The increased release at a lower pH is attributable to protonation of amino groups, which induces electrostatic repulsion and loosens the hydrogel network. Collagenase further accelerates release through selective enzymatic degradation, producing nearly complete release within 72 h. These results indicate a release profile that is both prolonged and tumor-responsive, aligning with the pathological microenvironment.

Biocompatibility was confirmed by cell viability assays, where blank Gelatin MA and PPy@GO hydrogels exhibited negligible cytotoxicity. Drug-loaded hydrogels preserved cytotoxic activity while reducing acute toxicity compared with free drugs, reflecting sustained release behavior. Notably, cisplatin and epirubicin exhibited synergistic effects, with the combination hydrogel producing significantly greater cytotoxicity than either monotherapy. Fluorescence imaging corroborated these findings, showing gradual uptake of hydrogel-encapsulated epirubicin with delayed nuclear localization relative to free drug, consistent with controlled release.

The photothermal component, PPy@GO, demonstrated strong NIR absorption and a stable heating performance, confirming successful PPy coating on GO. TEM revealed PPy deposition on GO sheets with lateral dimensions of 200–500 nm, a size range reported to undergo rapid hepatic metabolism. In vitro heating tests confirmed rapid temperature increases to ≥69 °C under NIR irradiation, with reproducibility across multiple cycles, indicating a stable photothermal capacity. In vitro cytotoxicity assays showed that P-gel under NIR irradiation significantly reduced cell viability, whereas blank gels and irradiation alone had no effect, confirming that heating was exclusively attributable to PPy@GO. In vivo studies verified blue-light-triggered gelation under physiological conditions, with FITC-labeled hydrogels forming stable solid implants upon irradiation. Thermal imaging further demonstrated effective photothermal heating of P-gel in vivo, with temperature elevations to about 53 °C localized to the irradiated site, levels sufficient to induce thermal cytotoxicity.

Therapeutic evaluation in tumor-bearing mice revealed that the combination hydrogel significantly suppressed tumor growth and extended survival compared with intravenous delivery, with minimal systemic toxicity, as evidenced by stable body weight. The addition of radiotherapy further enhanced efficacy, while the integration of chemo-, photothermal-, and radiotherapy modalities (CEP-gel + Laser + RT) produced the most pronounced outcomes, including complete tumor regression in some animals and the highest survival rates. These results highlight the capacity of this hydrogel system to integrate multiple therapeutic mechanisms within a single local platform, maximizing antitumor efficacy while minimizing systemic side effects.

Although we have demonstrated that our system can successfully perform in situ gelation and controlled release of drugs, combined with PTT to eradicate GBM in vitro and in vivo, this study also has following limitations. In this study, we used U87-MG and MBR614-2 cell lines as our in vitro model, but the effect of chemotherapy drugs may vary between different cells. Therefore, the dosage we used in this study may not directly reflect the real dosage that can be used in clinical applications. In the animal model, we used a cell line inoculation tumor model in C57BL/6 mice. The microenvironment of tumors in the animal model may not be exactly the same as that in real human tumors. Therefore, the dosage and gelation conditions may be further optimized for real human tissue. The other limitation is the mechanical strength of the gel. In this study, we used Gel MA as the base gelation material for in situ gelation. It released the drug and degraded within several days. For the long-term slow release of drugs, such as drugs for Alzheimer disease, or slower release of chemotherapy drugs in the brain, the long-term mechanical strength and stability should be optimized to fit the specific applications.

Taken together, this study demonstrates a proof-of-concept in situ gelation Chemo-PTT combination delivery system. We have proved that Gelatin MA hydrogels incorporating PPy@GO, cisplatin, and epirubicin represent a versatile and biocompatible platform for multimodal cancer therapy. The system provides rapid in situ gelation, sustained and tumor-responsive drug release, a robust photothermal performance, and synergy with radiotherapy. Such characteristics suggest considerable translational potential for localized treatment of brain tumors and other solid malignancies.

## 5. Conclusions

This study successfully synthesized methacrylated gelatin (Gelatin MA) through methacryloyl modification and achieved rapid blue-light-initiated crosslinking in the presence of VA-086, resulting in a hydrogel drug delivery system with convenient handling and fast gelation. Both blank Gel (without PPy@GO) and P-gel (containing PPy@GO but without laser irradiation) demonstrated negligible cytotoxicity, confirming the high biocompatibility and low inherent toxicity of the Gelatin MA, PPy@GO, and VA-086 crosslinking process.

Encapsulation of cisplatin and epirubicin within Gelatin MA enabled sustained release under physiological conditions while preserving drug bioactivity. Fluorescence imaging and cytotoxicity assays confirmed that the released drugs were effectively internalized by cells and retained their antitumor activity. Similarly, PPy@GO maintained strong photothermal properties after encapsulation, efficiently absorbing NIR light and generating cytotoxic heating effects. In vitro experiments demonstrated that drug-loaded gels (C-gel, E-gel, and CE-gel) exhibited significant cytotoxicity, although less acute than the equivalent free-drug formulations, consistent with controlled release. Fluorescence imaging further supported gradual intracellular accumulation of epirubicin released from hydrogels.

In vivo experiments confirmed that the hydrogel system could be injected and crosslinked under complex physiological conditions, with encapsulated PPy@GO exhibiting robust photothermal heating sufficient to induce tumor cell damage. Most notably, CEP-gel combined with photothermal therapy and radiotherapy achieved potent tumor suppression, inducing marked tumor regression without significant systemic toxicity. Treatment extended animal survival by more than 22 days compared with controls, underscoring the therapeutic advantage of this multimodal platform.

Collectively, these findings establish Gelatin MA hydrogels incorporating PPy@GO, cisplatin, and epirubicin as a versatile, biocompatible, and effective strategy for localized cancer therapy. The system integrates chemotherapy, photothermal therapy, and radiotherapy within a single injectable platform, providing sustained release, controlled activation, and synergistic efficacy. These results highlight the translational potential of this hydrogel-based delivery system for improving clinical outcomes in the treatment of solid tumors.

## Figures and Tables

**Table 1:** Mixing ratios of Gelatin MA, drug, photothermal material, and photoinitiator.

	Gelatin MA 250 mg/mL	EPI 80 mg/mL	Cisplatin 5 mg/mL	PPy@GO 0.5 mg/mL	D.I. H_2_O	VA-086 200 mg/mL
Gel only	144				26	30
C-gel	144		14		12	30
E-gel	144	6.6			19.4	30
CE-gel	144	6.6	14		5.4	30
P-gel	144			5	21	30
CEP-gel	144	6.6	14	5	0.4	30

Unit: μL.

**Table 2:** First and second degradation temperatures from thermogravimetric analysis (TGA) of Gel, Gelatin MA, and Gelatin.

Gelatin	Gelatin MA	Gel
71.6	102.7	163.4
338.4	340.4	341.7

Unit: °C.

## Data Availability

Data will be made available on request.

## Supplementary Materials

The following supporting information can be downloaded at: https://www.mdpi.com/article/10.3390/pharmaceutics17101353/s1, A. Supporting Methods including I. Materials list and II. II. Detailed Animal Experiments and Procedures (Methods S1–S9) detailly described the S1. Ethical Statement, S2. Experimental Animals and Husbandry, S3. Study Design and Sample Size, S4. Randomisation and Blinding, S5. General Procedures and Animal Welfare, S6. Specific Experimental Protocols, S7. Outcome Measures and Data Collection, S8. Euthanasia and Tissue Collection, and S9. Statistical Methods. B. Supporting Results including: Figure S1. Image for Gel-MA (a) before and (b) after gelation. Figure S2. Temperature variation curves (ΔT = T_gel_ − T_control_) of Gel and P-gel after subcutaneous gelation and 5 min irradiation with an 808 nm near-infrared laser in vivo. Figure S3. Image showing FITC-gel formation with (a,b) or without (c,d) in vivo blue light gelation. FITC-labeled hydrogel visualized under blue-light excitation (b,d).
