# Supplementary material for: Gelatin-Based Rapid Blue Light-Irradiation In Situ Gelation Hydrogel Platform for Combination Therapy in Brain Tumors
Source: Pharmaceutics. 2025 Oct 20;17(10):1353. doi: 10.3390/pharmaceutics17101353 (PMC12567308; doi:10.3390/pharmaceutics17101353)
Supplement: Supplementary file 1 [file pharmaceutics-17-01353-s001.zip › pharmaceutics-3870598-supplementary.pdf]

Supplementary materials

Gelatin-Based Rapid Blue Light-Irradiation In Situ Gelation Hydrogel Platform for Combination Therapy in Brain Tumors

Chiung-Yin Huang[1,†], Hung-Wei Yang[2,3,†], Hung-Chun Wang[4], Chia-Yu Hsu[5], Kuo-Chen Wei[1,6,7], Pin-Yuan Chen[1,7,8], Hao-Han Pang[5,*]*

[1]Neuroscience Research Center, Chang Gung Memorial Hospital, Linkou, Taoyuan 33305, Taiwan;

[2]Department of Biomedical Engineering, National Cheng Kung University, Tainan 70101, Taiwan;

[3]Medical Device Innovation Center, National Cheng Kung University, Tainan 70101, Taiwan

[4]Institute of Medical Science and Technology, National Sun Yat-sen University, Kaohsiung 80424, Taiwan;

[5]Center for Drug Research and Development, Collage of Human Ecology, Chang Gung University of Science and Technology, Taoyuan 33303, Taiwan;

[6]Department of Neurosurgery, Chang Gung Memorial Hospital, Linkou, Taoyuan 33305, Taiwan

[7]School of Medicine, Chang Gung University, Guishan, Taoyuan 33302, Taiwan

[8]Department of Neurosurgery, Chang Gung Memorial Hospital, Keelung 20401, Taiwan

[*]Correspondence: pinyuanc@cgmh.org.tw (P.-Y.C.); hhpang@mail.cgust.edu.tw (H.-H.P.)

[†]These authors contributed equally to this work.

Keywords: glioblastoma; GBM; combination therapy; hydrogel; photothermal therapy

A. Supporting methods

I. Materials List

The materials used in this study and their resource information are described as following:

1. 2,3-Bis(2-methoxy-4-nitro-5-sulfophenyl)-2H-tetrazolium-5-carboxanilide inner salt (XTT Salt) (SIGMA-ALDRICH, CAS: 1110-72-31-2, USA)

2. 2-2'-azobis[2-methyl-n-(2-hydroxyethyl)propionamide] (Wako Pure Chemicals, CAS : 61551-69-7, USA)

3. Bovine Serum Albumin (SIGMA-ALDRICH , CAS: 9048-46-8, USA)

4. Calcium Chloride, Anhydrous (J.T. Baker, CAS: 10043-52-4, Japen)

5. Cis-diamminedichloridoplatinum(II) (Cisplatin) (Fresenius KABI, CAS: 15663-27-1, India)

6. Collagenase from Clostridium histolyticum (SIGMA-ALDRICH, C0130, USA)

7. Dimethyl Sulfoxide (SIGMA-ALDRICH, CAS: 67-68-6, USA)

8. Dimethylformamide (SIGMA-ALDRICH, CAS: 68-12-2, USA)

9. Dulbecco's Modified Eagle Medium (DMEM), High Glucose with Phenol Red (GenedireX, LOT: GL17013, Taiwan)

10. Epirubicin Hydrochloride (USP, CAS:56390-09-1, Ukraine)

11. Ethanol (TAIWAN BURNETCT, CAS:64-17-5, Taiwan)

12. Fetal Bovine Serum (FBS) (Gibco, LOT: 1420798, USA)

13. Fluorescein isothiocyanate (FITC) (CAS: 27072-45-3)

14. Gelatin from Porcine Skin (SIGMA-ALDRICH, CAS: 9000-70-8, USA)

15. Graphite (SIGMA-ALDRICH, CAS: 17782-42-5, USA)

16. Hoechst 33342 (SIGMA-ALDRICH, CAS: 23491-52-3, USA)

17. Hydrochloric Acid (SIGMA-ALDRICH, CAS: 7647-01-0, Austria)

18. Methacrylic Anhydrous (SIGMA-ALDRICH, CAS: 760-93-0, USA)

19. Methanol (Macron, CAS: 67-56-1, USA)

20. *o*-Phenylenediamine (Alfa Aesar, CAS: 95-14-5, USA)

21. Penicillin (Gibco, CAS: 69-57-8, USA)

22. Polyvinyl Alcohol (SIGMA-ALDRICH, CAS: 9002-89-5, USA)

23. Potassium Chloride (SIGMA-ALDRICH, CAS: 7447-40-7, USA)

24. Potassium Phosphate Monobastic (SIGMA-ALDRICH, CAS: 7778-77-0, USA)

25. Pyrrole (ALDRICH, CAS: 109-97-7, USA)

26. Rhodamine B (SIGMA-ALDRICH, CAS: 81-88-9, USA)

27. Sodium Chloride (J.T. Baker, CAS: 1310-73-2, USA)

28. Sodium Phosphate, Dibastic, Anhydrous (J.T. Baker, CAS: 7558-79-4, India)

29. Streptomycin Sulfate (Carbosynth, CAS: 3810-74-0, USA)

30. Sulfuric acid (SIGMA-ALDRICH , CAS: 7664-93-9 , USA)

31. Sulfuric acid (SIGMA-ALDRICH, CAS: 7664-93-9, USA)

32. Trypsin-EDTA (Gibco, CAS: 9002-07-7, Canada)

II. Detailed Animal Experiments and Procedures

S1. Ethical Statement

All animal experiments in this study were conducted in accordance with the guidelines from the Institutional Animal Care and Use Committee (IACUC) of Chang Gung University. The experimental protocol was approved under the protocol code CGU110-034 (valid from July 2021 to June 2024).

S2. Experimental Animals and Husbandry

- **Species and Strain:** C57BL/6 mice were used in this study.
- **Source:** All animals were purchased from BioLASCO, Taiwan, a company accredited by the Association for Assessment and Accreditation of Laboratory Animal Care (AAALAC) International.
- **Animal Details:** Unless otherwise specified, male C57BL/6 mice, approximately 6-8 weeks old, were used for the experiments.
- **Housing Conditions:** Animals were housed in individually ventilated cages (IVCs) with 4 mice per cage. They were maintained under standard laboratory conditions: a temperature of 22±2∘C, humidity of 55±10%, and a 12-hour light/12-hour dark cycle. Standard chow and sterile water were available ad libitum. Standard environmental enrichment was provided to enhance animal welfare.

S3. Study Design and Sample Size

- **Study Design:** This research involved three main in vivo experiments. Each experiment included a control group and one or more treatment groups for comparison. The experimental unit was a single mouse.
- **Sample Size:** In the tumor suppression study, each treatment and control group consisted of 4 animals (n=4). The sample size was determined based on previous experience with similar studies to ensure sufficient statistical power for evaluating treatment efficacy, particularly for survival curve analysis.

S4. Randomisation and Blinding

- **Randomisation:** After successful tumor cell inoculation and when tumors

reached a volume of approximately 20 mm^3, mice were stratified by body weight and tumor volume. They were then randomly assigned to treatment and control groups using a random number generator to ensure no significant initial differences between groups.

- **Minimization of Potential Confounders:** Animals from different experimental groups were co-housed in various cages, and the order of daily measurements and treatments was randomized to minimize potential bias arising from cage location or handling order.
- **Blinding:** Due to the nature of certain treatments (e.g., laser irradiation), the investigators administering the treatments could not be blinded. However, the personnel responsible for tumor volume measurement and data analysis were blinded to the group allocations to reduce observer bias.

S5. General Procedures and Animal Welfare

- **Anesthesia:** All surgical or procedural interventions requiring anesthesia (e.g., tumor inoculation, imaging) were performed using 2% isoflurane inhalation, with a gas flow rate of 800 ml/min. To prevent corneal drying during anesthesia, an ophthalmic gel (Gendermin 3 mg/g) was applied to the animals' eyes.
- **Post-operative Care and Monitoring:** Following any procedure, animals were placed on a warming pad or under a heat lamp until fully recovered, after which they were returned to their home cages. Animals were monitored daily for general activity, food and water intake, and body weight changes.
- **Pain Management:** To minimize pain and distress, prophylactic analgesics were administered after surgical procedures. During the tumor growth period, pain assessments were conducted twice weekly. If an animal's pain score reached a predetermined threshold, carprofen (5 mg/kg) was administered subcutaneously daily for pain relief. If pain was not alleviated after three consecutive days of analgesic treatment, the animal was humanely euthanized.
- **Humane Endpoints:** Clear humane endpoints were established for this study. The experiment was terminated for an animal, and it was humanely euthanized

if any of the following conditions were met:

- Tumor volume exceeded 2,000 mm^3.
- Body weight loss exceeded 20% of the initial weight within 3-4 days.
- The animal exhibited signs of severe distress, such as a ruffled coat, significantly reduced activity, refusal to eat or drink, or abnormal behaviors like self-mutilation.

S6. Specific Experimental Protocols

S6.1. *In Vivo* Gelation of Hydrogel

A 40 μL solution of Gelatin MA, mixed with a photosensitizer and FITC fluorescent dye, was injected subcutaneously into the dorsal region of C57BL/6 mice anesthetized with isoflurane. Immediately following injection, the site was irradiated with a 395 nm blue light at close range for 10 minutes to induce gelation. Control mice received the same injection without light exposure. Subsequently, the tissue at the injection site was excised, and FITC fluorescence was visualized and photographed under blue light excitation.

S6.2. *In Vivo* Photothermal Effect Study

Hydrogel formulations (Gel and P-gel, 40 μL) containing a photosensitizer were subcutaneously injected into anesthetized C57BL/6 mice. The site was then irradiated with 395 nm blue light for 10 minutes to ensure gelation. After the animal and environment returned to room temperature, an 808 nm near-infrared (NIR) laser (75% power output) was used to irradiate the gel site for 5 minutes. A FLIR infrared thermal camera was used to record the local temperature changes during this period.

S6.3. *In Vivo* Tumor Suppression Study

U87-MGFL tumor cells (2×10^5 cells) were subcutaneously inoculated into the ventral side of C57BL/6 mice. On the fifth day, when tumor volumes reached approximately 20 mm^3, the initial tumor volume and body weight were recorded, and treatments were initiated.

- **Treatment Groups:**
  - **Hydrogel Groups (P-gel, CEP-gel):** A 40 μL volume of hydrogel was

injected into the tumor center and periphery, followed by 10 minutes of 395 nm blue light irradiation.

- **Free-Drug Groups (Freeform-C, Freeform-E):** An equivalent drug concentration in solution was injected at the same sites, without light irradiation.
- **Laser Group:** After injection of P-gel and light-induced gelation, the tumor site was irradiated with an 808 nm NIR laser for 5 minutes.
- **Radiotherapy (RT) Group:** Mice received a radiation dose of 5 Gy.

- **Data Collection:** Tumor dimensions (length, width, height) were measured with calipers, and body weight was recorded every three days.

S7. Outcome Measures and Data Collection

- **Primary Outcome Measures:** The primary outcome measures for this study were **animal survival** and **change in tumor volume**, which were used to assess the efficacy of the different therapeutic approaches.
- **Secondary Outcome Measures:** These included changes in animal body weight and local temperature changes during photothermal therapy.
- **Calculation Formulas:**
    - Tumor Volume Calculation = Tumor length × Tumor width × Tumor height2   (unit: mm^3)
    - Relative Tumor Volume Calculation =
      Tumor volume on measurement day-
      Initial tumor volumeInitial tumor volume x 100%
    - Relative Body Weight Calculation =
      Body weight on measurement day-Initial body weightInitial body weight x 100%
- **Data Exclusion Criteria:** During the experiment, data from all animals were included in the analysis unless an animal met the criteria for a humane endpoint. No other pre-established criteria for data exclusion were set.

S8. Euthanasia and Tissue Collection

At the conclusion of the experiment or upon reaching a humane endpoint, animals were euthanized by carbon dioxide (CO_2) inhalation or by cervical dislocation following over-anesthetization with isoflurane. Tumor and other tissue samples were then collected as required for subsequent analysis. Animal carcasses were disposed of professionally through the university's animal care facility.

S9. Statistical Methods

All statistical analyses were performed using GraphPad Prism software. Data are presented as mean ± standard deviation (SD). Comparisons between groups were made using a one-way analysis of variance (ANOVA) or a t-test. Survival curves were generated using the Kaplan-Meier method and analyzed with the log-rank test. A p-value of less than 0.05 was considered statistically significant in all analyses.

B. Supporting Results

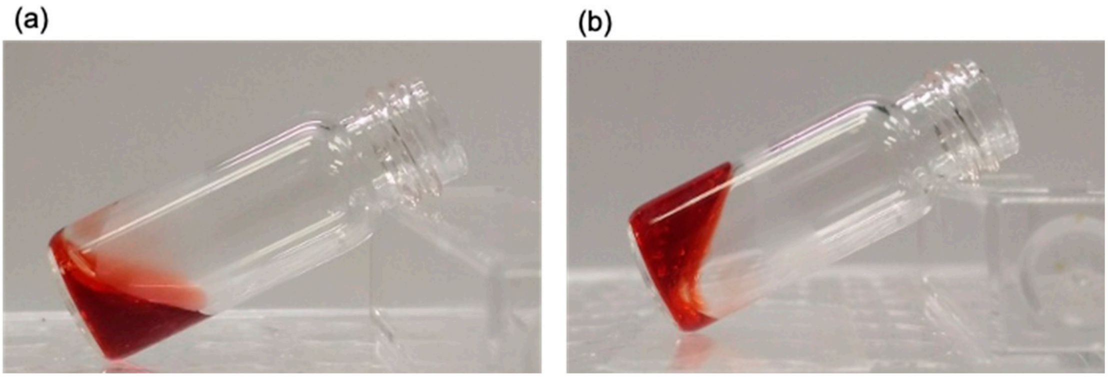

Figure S1. Image for Gel-MA (a) before and (b) after gelation.

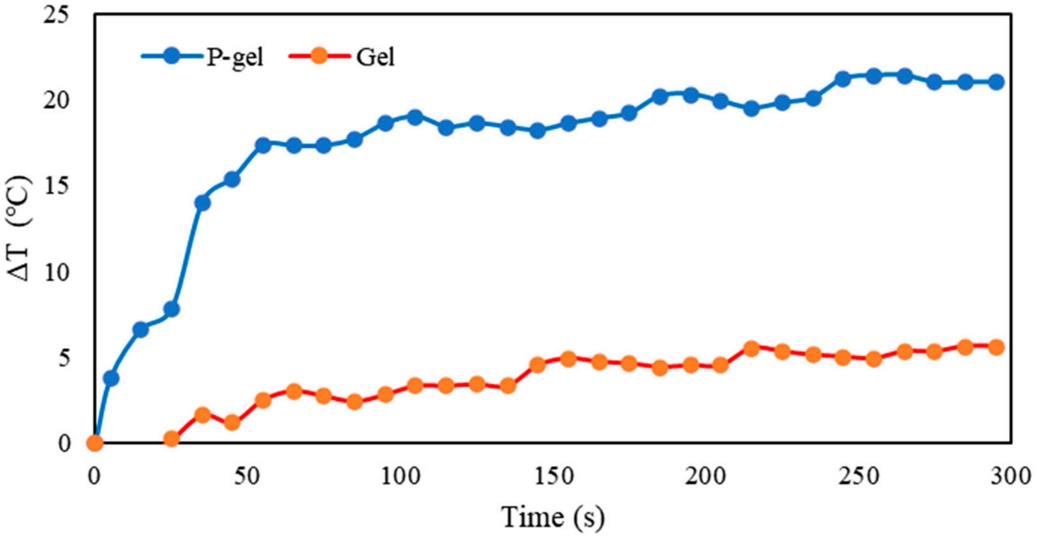

Figure S2. Temperature variation curves ($\Delta T = T_{gel} - T_{control}$) of Gel and P-gel after subcutaneous gelation and 5 min irradiation with an 808 nm near-infrared laser in vivo.

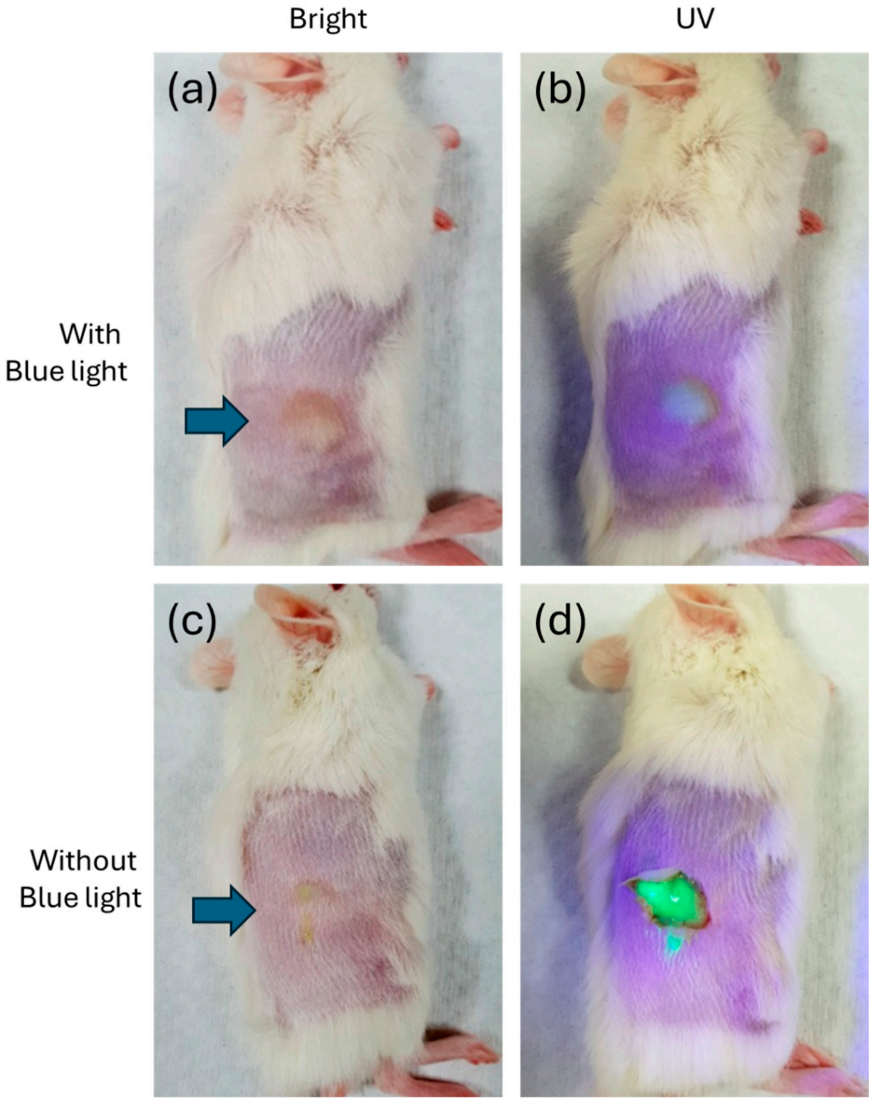

Figure S3. Image showing FITC-gel formation with (**a, b**) or without (**c, d**) in vivo blue light gelation. FITC-labeled hydrogel visualized under blue-light excitation (**b, d**).
